# Aberrant expression of NKL homeobox genes HMX2 and HMX3 interferes with cell differentiation in acute myeloid leukemia

**DOI:** 10.1371/journal.pone.0240120

**Published:** 2020-10-13

**Authors:** Stefan Nagel, Claudia Pommerenke, Corinna Meyer, Roderick A. F. MacLeod, Hans G. Drexler

**Affiliations:** Department of Human and Animal Cell Lines, Leibniz-Institute DSMZ–German Collection of Microorganisms and Cell Cultures, Braunschweig, Germany; Emory University, UNITED STATES

## Abstract

The NKL-code describes normal expression patterns of NKL homeobox genes in hematopoiesis. Aberrant expression of NKL homeobox gene subclass members have been reported in several hematopoietic malignancies including acute myeloid leukemia (AML). Here, we analyzed the oncogenic role of the HMX-group of NKL homeobox genes in AML. Public expression profiling data–available for HMX1 and HMX2—indicate aberrant activity of HMX2 in circa 2% AML patients overall, rising to 31% in those with KMT2A/MLL rearrangements whereas HMX1 expression remains inconspicuous. AML cell lines EOL-1, MV4-11 and MOLM-13 expressed both, HMX2 and neighboring HMX3 genes, and harbored KMT2A aberrations, suggesting their potential functional association. Surprisingly, knockdown experiments in these cell lines demonstrated that KMT2A inhibited HMX2/3 which, in turn, did not regulate KMT2A expression. Furthermore, karyotyping and genomic profiling analysis excluded rearrangements of the HMX2/3 locus in these cell lines. However, comparative expression profiling and subsequent functional analyses revealed that IRF8, IL7- and WNT-signalling activated HMX2/3 expression while TNFa/NFkB- signalling proved inhibitory. Whole genome sequencing of EOL-1 identified two mutations in the regulatory upstream regions of HMX2/3 resulting in generation of a consensus ETS-site and transformation of a former NFkB-site into an SP1-site. Reporter-gene assays demonstrated that both mutations contributed to HMX2/3 activation, modifying ETS1/ELK1- and TNFalpha-mediated gene regulation. Moreover, DMSO-induced eosinophilic differentiation of EOL-1 cells coincided with HMX2/3 downregulation while knockdown of HMX2 induced cell differentiation, collectively supporting a fundamental role for these genes in myeloid differentiation arrest. Finally, target genes of HMX2/3 were identified in EOL-1 and included suppression of differentiation gene EPX, and activation of fusion gene FIP1L1-PDGFRA and receptor-encoding gene HTR7, both of which enhanced oncogenic ERK-signalling. Taken together, our study documents a leukemic role for deregulated NKL homeobox genes HMX2 and HMX3 in AML, revealing molecular mechanisms of myeloid differentiation arrest.

## Introduction

Acute myeloid leukemia (AML) is a malignant disease characterized by clonal expansion of undifferentiated myeloid precursors. AML is the most common acute leukemia in the elderly and classified according to chromosomal aberrations [1]. The advent of next generation sequencing technologies has boosted our understanding of molecular alterations and their consequences in myeloid malignancies [2]. Accordingly, a recent classification scheme for AML combines driver mutations and cytogenetics, highlighting causative genomic changes [3]. Nevertheless, certain key oncogenic fusion genes retain their peak diagnostic and therapeutic significance. The KMT2A/MLL gene encodes a histone methyltransferase which is aberrantly fused to several partner genes by specific recurrent chromosomal translocations, defining an AML subgroup [1, 4]. In addition to gene fusions, KMT2A gene aberrations comprise amplifications and partial tandem duplications [5]. KMT2A methylates histone H3, generating H3K4me3 which is associated with gene activation [6]. Key oncogenic KMT2A-targets are transcription factors encoded by the HOXA homeobox gene cluster predominantly expressed at early stages of hematopoiesis [7].

Primary eosinophilia represents an additional subgroup of myeloid neoplasms which is further categorized according to the presence of particular fusion genes including PCM1-JAK2 or those involving PDGFRA, PDGFRB or FGFR1 [1]. Seven partner genes of PDGFRA have been described hitherto, including FIP1L1 and FOXP1 [8]. The eosinophilic AML cell line EOL-1 expresses the fusion gene FIP1L1-PDGFRA and serves as a unique model for revealing leukemic functions of this oncogene [9]. In addition, EOL-1 contains a partial tandem duplication of the KMT2A gene [10], thus combining two genetic characteristics from different subgroups of myeloid malignancies. Its property of differentiating towards eosinophils after stimulation warrants this cell line as a practical model of deregulated differentiation in AML [11].

Transcription factors encoded by homeobox genes widely impact developmental processes both during embryogenesis and in the adult. Accordingly, their deregulation contributes to the generation of cancer including leukemia [12, 13]. NKL homeobox genes represent a subclass comprising 48 members in humans and contains several developmental master genes like the heart-organizer NKX2-5 and the spleen-organizer NKX3-2 [14, 15]. We have studied the physiological expression of all NKL subclass members in lymphoid and myeloid hematopoiesis and identified specific patterns which we have termed the NKL-code [16–18]. In myelopoiesis this code involves six genes: namely DLX2, HHEX, HLX, HMX1, NKX3-1 and VENTX [18]. Deregulated NKL homeobox genes in myeloid malignancies include both NKL-code members (e.g. DLX2 and NANOG) and ectopically activated non-members (e.g. HMX2 and NKX3-2) [18]. In AML, NKL homeobox genes DLX1 and DLX2 are aberrantly activated via the ERK-pathway, and NANOG via stem cell factors STAT3 and TET2 and the NOTCH-pathway [18, 19], thus illustrating a wide diversity of deregulation modes.

In this study, we investigated deregulated NKL homeobox genes of the HMX-group which comprises HMX1, HMX2 and HMX3. These three genes are normally expressed in the craniofacial region of the embryo. HMX1 is active in the second arch, the developing eye, and controls the development of the outer ear [20, 21]. HMX2 and HMX3 are predominantly coexpressed in the hypothalamus and in the otic region controlling development of the inner ear [20, 22]. In the adult, HMX1 is expressed in erythropoiesis while HMX2 and HMX3 are silent during the complete course of hematopoiesis [18].

Here, we examined aberrant activities of NKL homeobox genes HMX2 and HMX3 in AML which were coexpressed in concert with KMT2A rearrangements. We revealed upstream factors and downstream effects including the disturbance of myeloid differentiation, highlighting their leukemic potential.

## Materials and methods

### Expression profiling and transcriptome analysis

Public expression profiling data from primary AML patient samples were generated using Affymetrix gene chips HG-U133-plus2 and drawn from Gene Expression Omnibus (GEO; www.ncbi.nlm.nih.gov/gds). Used datasets were as follows: GSE15434 [23], GSE61804 [24], GSE21261 [25] and GSE19577 [26]. Expression profiling analysis of AML cell lines employed dataset GSE59808, and of primary myeloid peripheral blood cells dataset GSE109348 [27]. Expression data were analyzed using the associated online tool GEO2R. After RMA-background correction and quantile normalization of the spot intensities data processing was performed via R/Bioconductor using limma and affy packages. Differentially expressed genes between HMX2/3 positive cell lines and negative controls were listed according moderated t-statistics (R, limma). To parse biological function 250 or 1000 differentially expressed genes were shortlisted. Subsequent gene-annotation enrichment analyses (GAEA) were performed using DAVID bioinformatics resources (www.david.ncifcrf.gov), to yield GO-terms and KEGG-pathways for the shortlisted gene-sets [28].

Transcriptome data from 100 leukemia/lymphoma cell lines (LL-100) were drawn from ArrayExpress (www.ebi.ac.uk/arrayexpress), using dataset E-MTAB-7721 [29]. RNA-seq data were mapped (STAR), counted (HTSeq) and normalized (DESeq2) as described previously [29]. Graphical presentations of the LL-100 normalized gene expression count data were performed using shinyNGS (https://github.com/pinin4fjords/shinyngs). According to the original publication, we set of the cut-off for positive gene expression at 500 normalized counts [29].

### Cell lines and treatments

AML-derived cell lines have been obtained from the DSMZ (German Collection of Microorganisms and Cell Lines, Braunschweig, Germany), a public, non-profit biological ressources center owned by the German government. Cell culture conditions, culture media and other relevant information on each cell line are provided in detail on the institute`s website at www.dsmz.de and described elsewhere [30]. EOL-1 and MV4-11 were kindly provided by Prof. Jun Minowada, Fujisaki Cell Center, Okayama, Japan. The cell lines are monitored and validated by a unique program of intensity and quality which is rigorously implemented for all cell lines like authentication, exclusion of cross-contamination, documentation of freedom from inadvertent mycoplasm and viral contamination. Cell stimulations were performed for 16 h by treatment with 20 ng/ml of the following recombinant human proteins (all obtained from R&D Systems, Wiesbaden, Germany): tumor necrosis factor alpha (TNFa), interleukin (IL)7, FLT3 ligand (LG), WNT3A and WNT5B. Pharmacological drugs and chemicals were obtained from Sigma (Taufkirchen, Germany) and used at the indicated concentrations: dimethyl sulfoxide (DMSO), ERK-inhibitor PD98059 (35 μM), PDGFRA-inhibitor dasatinib (100 μM), HTR7-activator LP211 (1 μM), HTR7-inhibitor methysergide maleate salt (MMS, 1 μM), NFkB-inhibitor (14 μM).

Gene specific siRNA oligonucleotides (Hs_MLL_1, Hs_HMX2_4, Hs_HMX3_4, Hs_IRF8_4, Hs_EPX_1) and AllStars negative Control siRNA (siCTR, #1027281) were purchased from Qiagen (Hilden, Germany). Expression constructs for ELK1 (SC116858), ETS1 (SC125359), EPX (RC218959), HMX2 (SC303658) and SP1 (SC101137) were purchased from Origene (Wiesbaden, Germany). SiRNAs (80 pmol) and expression constructs/vector controls (2 μg) were transfected into 1x10^6^ cells by electroporation using the EPI-2500 impulse generator (Fischer, Heidelberg, Germany) at 350 V for 10 ms in the presence of medium and serum. Transfected cells were cultivated by addition of 1 ml serum-containing medium and harvested after 20 h cultivation.

For cytological analyses cell lines were stained with Giemsa-May-Grünwald as follows: cells were spun onto microscope slides and fixed for 5 min with methanol. Subsequently they were stained for 3 min with May-Grünwald´s eosine-methylene blue solution modified (Merck, Darmstadt, Germany) diluted in Titrisol (Merck), and for 15 min with Giemsa azur eosin methylene blue solution (Merck). Images were captured with an AXIO Scope A1 microscope using AxioCam MRc5 and software AxioVision 4.7 (Zeiss, Göttingen, Germany).

### Chromosomal and genomic analyses

Cytogenetic analysis was performed as described previously [31]. For genomic profiling genomic DNA of AML cell lines was prepared by the Qiagen Gentra Puregene Kit (Qiagen). Labelling, hybridization and scanning of HD Cytoscan arrays was performed at the Genome Analytics Facility, Helmholtz Centre for Infection Research (Braunschweig, Germany), using HD arrays according to the manufacturer´s protocols (Affymetrix/Thermo Fisher, Darmstadt, Germany). Data were visualized and interpreted using the Chromosome Analysis Suite software version 2.0.1.2 (Affymetrix).

### Polymerase Chain-Reaction (PCR) analyses

Total RNA was extracted from cell line samples using 1 ml TRIzol reagent (Invitrogen, Darmstadt, Germany) and 0.2 ml chloroform. Primary human RNA was obtained commercially. We used total RNA of basophils, eosinophils, neutrophils, and monocytes, all obtained from 3H Biomedical AB (Uppsala, Sweden). cDNA was synthesized by random priming from 5 μg RNA using Superscript II (Invitrogen). Real-time quantitative (RQ)-PCR analysis was performed with the 7500 Real-time Fast System, using commercial buffer and primer sets (Thermo Fisher Scientific). The following primer sets were used: CD11B (Hs00167304_m1), DLX2 (Hs00207788_m1), EPX (Hs00946094_1), ELK1 (Hs00901847_m1), ETS1 (Hs00428293_m1), HMX2 (Hs01394375_m1), HMX3 (Hs1392772_m1), HTR7 (Hs04194798_s1), IL7R (Hs00902334_m1), IRF8 (Hs00175238_m1), KMT2A (Hs00610538_m1), PDGFRA (Hs00998018_m1), STAT5A (Hs00559638_m1), and TBP (Hs00427620_m1). The temperature protocol was as follows: 15 s 90°C, 1 min 60°C, 40 cycles. Analysis and normalization of expression levels was performed using the ddCT-method and the transcript of TATA box binding protein (TBP). The obtained values were indicated as fold expression in relation to one selected sample which was set to 1. Experiments were performed twice and subsequent quantitative analyses in triplicate. Standard deviations are presented in the figures as error bars. Statistical significance was assessed by t-Test and derived p-values were indicated by asterisks (* p<0.05, ** p<0.01, *** p<0.001, n.s. not significant).

For detection of FIP1L1-PDGFRA fusion transcripts and FIP1L1 controls we performed reverse transcription (RT)-PCR, using the following oligonucleotides: FIP1L1-forward 5´-ACCTGGTGCTGATCTTTCTGAT-3´, FIP1L1-reverse 5´-CAGCTCCTGGAGGGAAAAAC-3´, and PDGFRA-reverse 5´-TGAGAGCTTGTTTTTCACTGGA-3´. For detection of KMT2A partial tandem duplication (PTD) we used the following oligonucleotides: KMT2A-forward1 5´-CCACTCCTAGTGAGCCCAAG-3´, KMT2A-reverse1 5´-GATGTTGCCTTCCACAAACG-3´, KMT2A-reverse2 5´-ACAGGCATGATGAACTGTCG-3´. For detection of KMT2A fusion transcripts we used the following oligonucleotides: KMT2A-forward2 5´-TCCAGAGCAGAGCAAACAGA-3´, AFF1-reverse 5´-CGTTCCTTGCTGAGAATTTG-3´, and KMT2A-forward3 5´-AGGACCGCCAAGAAAAGA-3´, MLLT3-reverse 5´- TACAGGCCTCTCCATTTCAG-3´. Oligonucleotides were purchased from Eurofins MWG (Ebersberg, Germany) and PCR products generated using taqpol (Qiagen) and thermocycler TGradient (Biometra, Göttingen, Germany). The temperature protocol was as follows: 1 min 90°C, 1 min 55°C, 2 min 72°C, 40 cycles. The products were analyzed by gel electrophoresis and documented with the Azure c200 Gel Imaging System (Azure Biosystems, Dublin, CA, USA).

### Sequencing

Genomic DNA was isolated from EOL-1 cells using the Gentra Puregene Kit (Qiagen). Whole genome sequencing of one sample from this cell line was conducted by Genewiz (Leipzig, Germany). Sequencing was performed using Illumina NovaSeq with 2x150 bp paired-end configuration with ≥80% of bases ≥Q30 and about 90 Gb coverage. The primary data are available at ENA (E-MTAB-9079). Mutation analysis by Genewiz included samtools (1.2), Isaac Aligner (04.17.06.15), Strelka Germline Variant Caller (2.8.0), and Annotation Dataset (84.24.38) resulting in >3.7 Mio SNVs for EOL-1, of which 22299 SNVs were located in coding regions. Sequencing data were visually analyzed using the Integrative Genomics Viewer obtained from the Broad Institute (www.software.broadinstitute.org).

Sanger sequencing of subcloned PCR products for IL7R was performed at Eurofins MWG. IL7R amplification was performed using oligonucleotides IL7R-forward 5´-TGCTCCAACCGGCAGCA-3´ and IL7R-reverse 5´-CCCTATGAATCTGGCAGTCC-3´ (Eurofins MWG). The PCR product (351 bp) was cloned into pGEM-T Easy (Promega, Walldorf, Germany) and indicated numbers of clones were sequenced. The identity and mutations of the sequences was analyzed using nucleotide BLAST (https://blast.ncbi.nlm.nih.gov/Blast.cgi).

### Reporter-gene assays

For creation of reporter gene constructs we combined a reporter with a regulatory genomic fragment derived from the flanking regions of HMX2, HMX3, EPX and HTR7. We cloned the genomic PCR products of the corresponding genomic regions (regulator) and of the HOXA9 gene, comprising exon1-intron1-exon2 (reporter), into the *Hind*III/*Bam*HI and *Eco*RI sites, respectively, of the expression vector pcDNA3 downstream of the CMV enhancer. The oligonucleotides used for the amplification of the regulators were obtained from Eurofins MWG. Their sequences were as follows: HMX2-for 5´-GCAAGCTTTGCCCGCCCACTGGGGCTAG-3´, HMX2-rev 5´-GGGGATCCGGCGGCGCGGAGGCGGCGCG-3´, HMX3-for 5´-TTAAGCTTCCAGATTTCCAAAGCAAAAATGGG-3´, HMX3-rev 5´-TAGGATCCTCGCCTAATTCAAGGCGCC-3´, EPX-for 5´-CAAAGCTTCGGCTGCTGTCCCAGGCCAGTG-3´, EPX-rev 5´-GGGGATCCTCCTGCAGCCATGGGTGGGATAG-3´, HTR7-for 5´-AGAAGCTTCCTGCTTCAGTATGTGCTCTGAAAC-3´, HTR7-rev 5´-TAGGATCCTCTCAATATCACAACCTTTTGCCTTC-3´. Introduced restriction sites used for cloning are underlined. The temperature protocol was as follows: 1 min 90°C, 1 min 55°C, 2 min 72°C, 40 cycles. The dimensions of the regulatory fragments were as follows: 202 bp (HMX3), 266 bp (EPX), 256 bp (HTR7). Constructs including mutations were validated by sequence analysis (Eurofins MWG). Transfections of plasmid-DNA into HELA or NIH-3T3 cells (DSMZ) were performed using SuperFect Transfection Reagent (Qiagen). The cells were harvested after 20 h incubation. Commercial HOXA9 (Hs00365956) and TBP assays (Hs00427620_m1 for HELA or Mm01277828_m1 for NIF-3T3) were used for RQ-PCR to quantify the spliced reporter-transcript, corresponding to the regulator activity (Thermo Fisher Scientific).

### Protein analyses

Western blots were generated by the semi-dry method. Protein lysates from cell lines were prepared using SIGMAFast protease inhibitor cocktail (Sigma). Proteins were transferred onto nitrocellulose membranes (Bio-Rad, München, Germany) and blocked with 5% dry milk powder dissolved in phosphate-buffered-saline buffer (PBS). The following antibodies were used: alpha-Tubulin (T6199, Sigma), HMX2 (NBP1-91997, Novus Biologicals, Abingdon, UK), HMX3 (LS-B8011, LSBio, Eching, Germany), ERK (sc-94, Santa Cruz Biotechnology, Heidelberg, Germany), phosphor (P)-ERK (sc-7383, Santa Cruz Biotechnology), EPX (LS-C354350, LSBio), HTR7 (LS-C358892, LSBio). For loading controls blots were reversibly stained with Poinceau (Sigma) and detection of alpha-Tubulin (TUBA) was performed thereafter. Secondary antibodies were linked to peroxidase for detection by Western-Lightning-ECL (Perkin Elmer, Waltham, MA, USA). Documentation was performed using the digital system ChemoStar Imager (INTAS, Göttingen, Germany).

## Results

### Deregulated HMX genes in AML

Aberrant expression of NKL homeobox gene HMX2 has been reported for subsets of AML patients [18]. Here, extended expression profiling analyses for HMX2 revealed aberrant gene activity in 5/251 (2.0%) AML patients with normal karyotype (GSE15434), 5/286 (1.7%) with normal or abnormal karyotype (GSE61804), and in 3/96 (3.1%) AML patients with myelodysplastic syndrome (MDS)-related changes and those whose disease was classified as not otherwise specified (GSE21261), indicating rather low incidence overall (**S1A–S1C Fig**). In contrast, frequent HMX2 expression was detected in 13/42 (31.0%) AML patients with KMT2A-rearrangements (GSE19577), demonstrating high incidence in this tumor subtype (**S1D Fig**). Furthermore, HMX2 activity was detected in 4/31 (12.9%) AML cell lines including EOL-1, MOLM-13 and MV4-11 (GSE59808), all of which carry KMT2A aberrations, coinciding with the picture in patient samples (**S1E Fig**). Of note, in these datasets HMX1 was not significantly expressed (**S1A–S1E Fig**) while HMX3 was not represented. Thus, although HMX1 is normally active in the erythroid branch of myelopoiesis these data provide little support for an oncogenic role in AML [18].

Next, to assess the potential role of aberrant HMX2 expression we performed comparative expression profiling analysis of these AML datasets and subsequently gene-annotation enrichment analyses (GAEA) of the top-250 differentially expressed genes. These data showed correlations of aberrant HMX2 activity with reported developmental processes operating in the inner ear and the brain/thalamus [22, 32], together with processes related to proliferation, innate immune response, and NFkB- and WNT-signalling (**S2A–S2E Fig**). Collectively, these findings serve to implicate HMX2 in deregulated developmental processes of myelopoiesis and of particular signalling-pathways in leukemic activation of HMX2.

**Fig 1:**
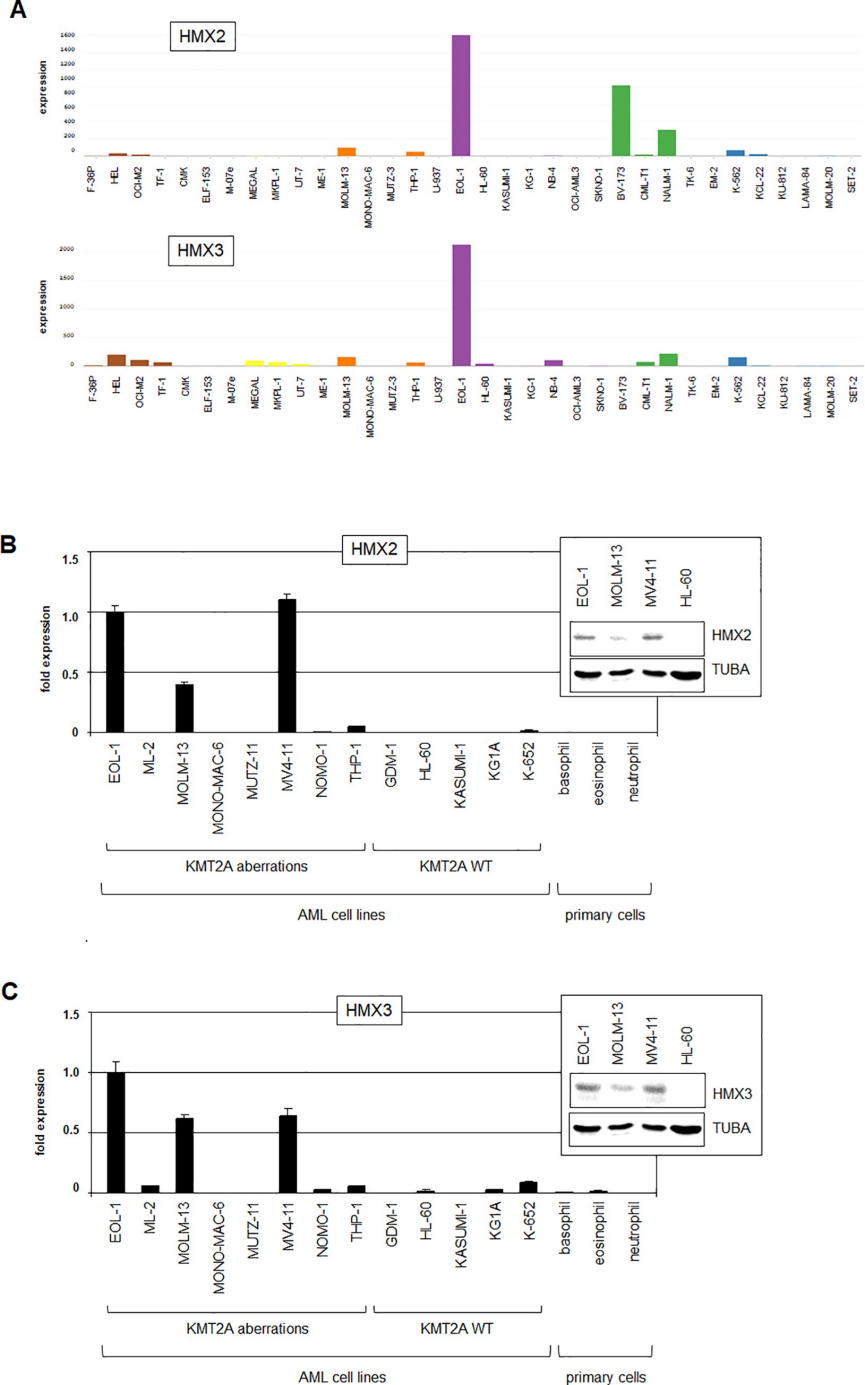
Expression of HMX genes in AML cell lines. (A) Gene expression values (DESeq2 normalized count data) are shown according to RNA-seq data from the LL-100 dataset for HMX2 (above) and HMX3 (below). The color-code indicates AML-subgroups: erythroid (brown), megakaryocytic (yellow), monocytic (orange), myelocytic (violet); CML-subtypes: myeloid (green), lymphoid (blue), myeloproliferativ neoplasm (just cell line SET-2). Of note, the scale illustrating the transcript amount is much smaller for HMX1. According to the setting of the cut-off at 500 normalized counts no cell line is positive for HMX1. The expression of HMX2 (B) and HMX3 (C) was analyzed in selected cell lines and primary samples from granulocytes by RQ-PCR and Western blot (inserts). TUBA served as loading control. Significant expression was detected in cell lines EOL-1, MOLM-13 and MV4-11 which all carry KMT2A aberrations. Of note, HL-60 expressed low levels of HMX3 but no HMX2 RNA. WT: wild type.

Analysis of RNA-seq dataset LL-100 (E-MTAB-7721) which contains 34 myeloid and 66 lymphoid malignant cell lines confirmed expression of HMX2 in EOL-1 which additionally expressed HMX3 while no cell line of this panel expressed HMX1 at meaningful levels (**S3 Fig, Fig 1A**). Of note, this panel does not contain the cell line MV4-11. Aberrant expression of HMX2 and/or HMX3 was also detected in a few lymphoid cell lines derived from B-cell precursor leukemia, chronic lymphoid leukemia and hairy cell leukemia (**S3 Fig**), also indicating deregulation of these genes in some B-lymphoid malignancies. RQ-PCR and Western blot analysis of selected AML cell lines demonstrated expression of both HMX2 and HMX3 in EOL-1, MOLM-13 and MV4-11 at the RNA and protein level (**Fig 1B and 1C**). Of note, HMX2 and HMX3 are genomic neighbors and coregulated under physiological conditions [32], hinting at a possible explanation for their leukemic coexpression.

Taken together, these data show expression of HMX2 and HMX3 in AML which was correlated with aberrant KMT2A activity and only rarely detected in other AML subtypes, suggesting regulatory connections. Additionally, NFkB- and WNT-signalling may play a role in HMX2/3 expression as well. In the following, we used three identified HMX2/3-positive AML cell lines, namely EOL-1, MOLM-13 and MV4-11, as models to characterize upstream factors and downstream effects of these deregulated NKL homeobox genes in this malignancy.

### Chromosomal and genomic analyses of AML cell lines

In lymphoid leukemia chromosomal rearrangements often mediate aberrant activation of particular oncogenes including members of the NKL homeobox gene subclass [33]. To look for chromosomal abnormalities, we performed cytogenetic analysis of three selected model AML cell lines—karyotypes were as follows:

EOL-1: 50(48–51)<2n> XY +4, del(4)(q12)x2, +6, +8, del(9)(q22), +19; MOLM-13: 51(48–52)<2n> XY, +8, +8, +8, del(8)(p1?p2?), ins(11;9)(q22.3;p23p24), +13; and MV4-11: 48(46–48)<2n> XY, t(4;11)(q21;q23), +8, +18, +19, -21. Thus, all three cell lines bore moderately hypodiploid karyotypes, including additional copies of chromosome 8, and solo translocations effecting recurrent gene fusions: FIP1L1-PDGFRA in EOL-1 via micro-deletion of 4q12; KMT2A-MLLT3 in MOLM-13 via insertion of 9p22/23 at 11q23; and KMT2A-AFF1 via t(4;11) in MV4-11 as described elsewhere [34, 35]. Accordingly, expression of the generated fusion genes was documented by RT-PCR analysis (**S4A Fig**). However, none bore visible rearrangements at 10q26 hosting HMX2 and HMX3. Additionally, we performed genomic profiling of EOL-1, MOLM-13 and MV4-11, revealing copy number alterations at particular chromosomal positions including gains at 11q13 and losses at 4q12 in EOL-1 (**S5 Fig**). However, these data also discounted aberrations at the locus of HMX2/3, excluding gene amplification as an oncogenic driver. The observed genomic gain at 11q23 in EOL-1 generated the reported partial tandem duplication (PTD) at KMT2A which was confirmed by RT-PCR (**S4B Fig**) [10].

**Fig 2:**
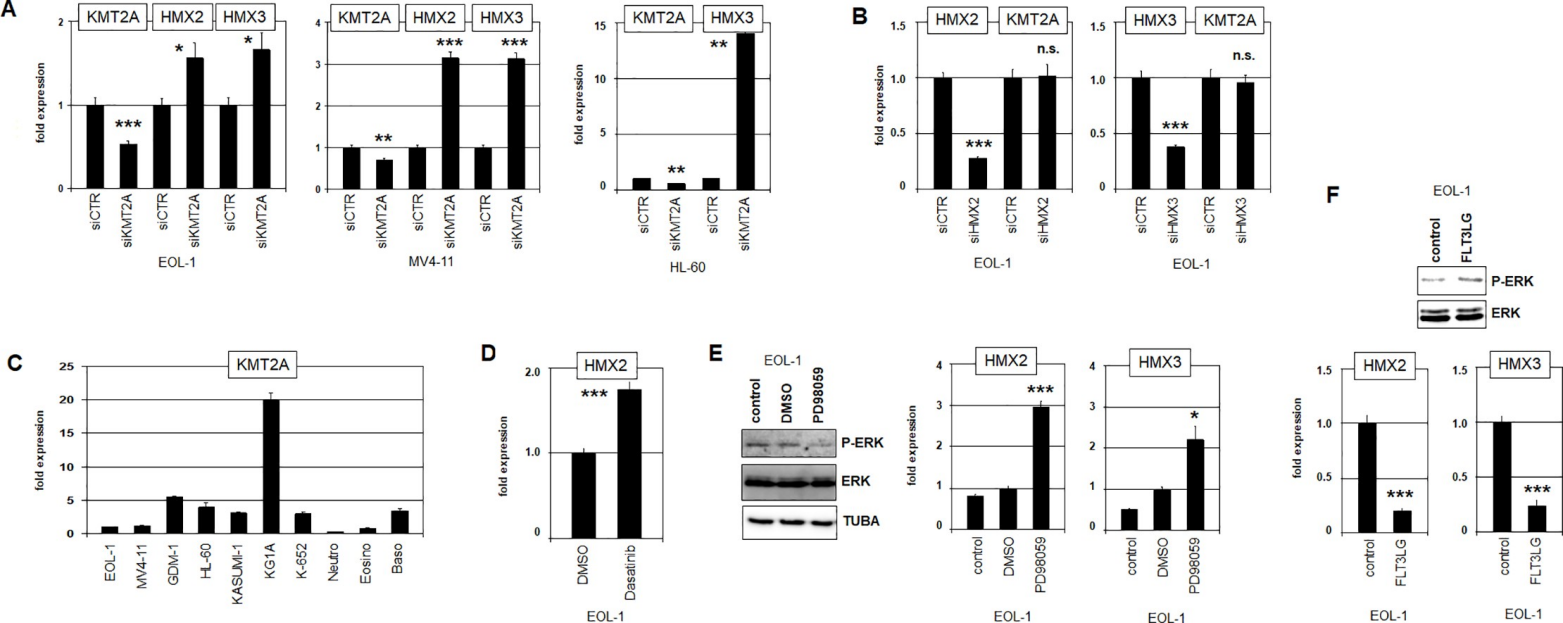
Regulation of HMX2 and HMX3 by KMT2A and ERK-signalling. (A) RQ-PCR analysis of EOL-1 (left) and MV4-11 (middle) treated for siRNA-mediated knockdown of KMT2A demonstrates raised expression levels of HMX2 and HMX3, indicating a repressive impact of KMT2A. This treatment resulted in enhanced expression of HMX3 in HL-60 cells which did not express HMX2 (right). Asterisks indicate calculated p-values obtained by t-Test analysis of controls (siCTR) and siRNA-targeted knockdown. (B) RQ-PCR analysis of KMT2A of EOL-1 cells treated for siRNA-mediated knockdown of HMX2 (left) and HMX3 (right) showed unaltered KMT2A expression levels, discounting any regulatory impact. (C) Quantification of KMT2A transcripts in AML cell lines and primary granulocytes showed low expression levels in EOL-1, MV4-11 and in eosinophils. (D) RQ-PCR analysis of HMX2 showed elevated expression levels in EOL-1 cells treated with PDGFRA-inhibitor dasatinib. Asterisks indicate calculated p-values obtained by t-Test analysis of controls (DMSO) and pharmacological treatments. (E) Treatment of EOL-1 cells with ERK-inhbitor PD98059 resulted in reduced levels of phospho-ERK as analyzed by Western blot (left), and in elevated transcript levels of HMX2 (middle) and HMX3 (right) as analyzed by RQ-PCR. (F) Treatment of EOL-1 cells with ERK-activator FLT3LG resulted in elevated levels of phosphorylated ERK (above) and in reduced transcript levels of HMX2 and HMX3 (below).

Then, we analyzed the potential mutual regulation of KMT2A and HMX2/3 which may underlie their transcriptional correspondence in patients and cell lines. Surprisingly, siRNA-mediated knockdown of KMT2A in EOL-1 and MV4-11 resulted in increased expression levels of HMX2 and HMX3 (**Fig 2A**). In addition, KMT2A-knockdown in HL-60 boosted expression of HMX3 while sparing HMX2 (**Fig 2A**). In contrast, siRNA-mediated knockdown of HMX2 and HMX3 left KMT2A expression levels unperturbed (**Fig 2B**). Thus, KMT2A operated as repressor for HMX2/3, both in wild type and rearranged contexts while HMX2/3 remained disengaged from KMT2A regulation. In accordance with these findings, the expression level of KMT2A was low in EOL-1, MV4-11 and MOLM-13 as demonstrated by RQ-PCR and transcriptome data (**Fig 2C, S4C Fig**). Moreover, primary eosinophils and eosinophilic cell line EOL-1 shared low KMT2A expression levels. Therefore, this physiological context may serve to promote aberrant activation of HMX2/3 in AML.

Furthermore, micro-deletion at 4q12 in EOL-1 generated the reported fusion gene FIP1L1-PDGFRA at the expense of CHIC2 (**S6A Fig**) [36]. Expression of this fusion gene was confirmed by RT-PCR (**S6B Fig**). However, inhibition of PDGFRA activity by dasatinib resulted in increased expression of HMX2 (**Fig 2D**), indicating an inhibitory effect. Functionally, PDGFRA mediates activation of the ERK-pathway in AML cells [37]. Consistent with this activity, inhibition of ERK-activity by PD98059 resulted in reduced ERK-phosphorylation and increased HMX2/3 expression, revealing suppressive impacts of this pathway on these genes (**Fig 2E**). Moreover, treatment of EOL-1 with ERK-activator FLT3LG resulted in reduced expression levels of HMX2/3 (**Fig 2F**).

Taken together, these analyses excluded genomic rearrangements of the NKL homeobox genes HMX2 and HMX3 and revealed an inhibitory impact of normal and rearranged KMT2A on their expression. In addition, aberrantly activated ERK-signalling also repressed HMX2/3 transcription. These results may suggest that their activators are not implicated in genomic aberrations.

### Identification of upstream factors mediating HMX2 and HMX3 expression

To identify activating regulators of HMX2 and HMX3 we analyzed expression profiling data of AML cell lines EOL-1 and MV4-11 in comparison to three HMX2/3-negative controls comprising cell lines GDM-1, HL-60 and KG-1. The top-1000 differentially expressed genes were analyzed by GAEA and conspicuous candidates selected (**S1 Table, S7 Fig**). This procedure revealed differences in signalling pathways (IL7, TNFa/NFkB, WNT) and transcription factor activities (ETS1, IRF8) all of which may contribute to HMX2/3 expression (**S1 Table, S7 Fig**).

**Fig 3:**
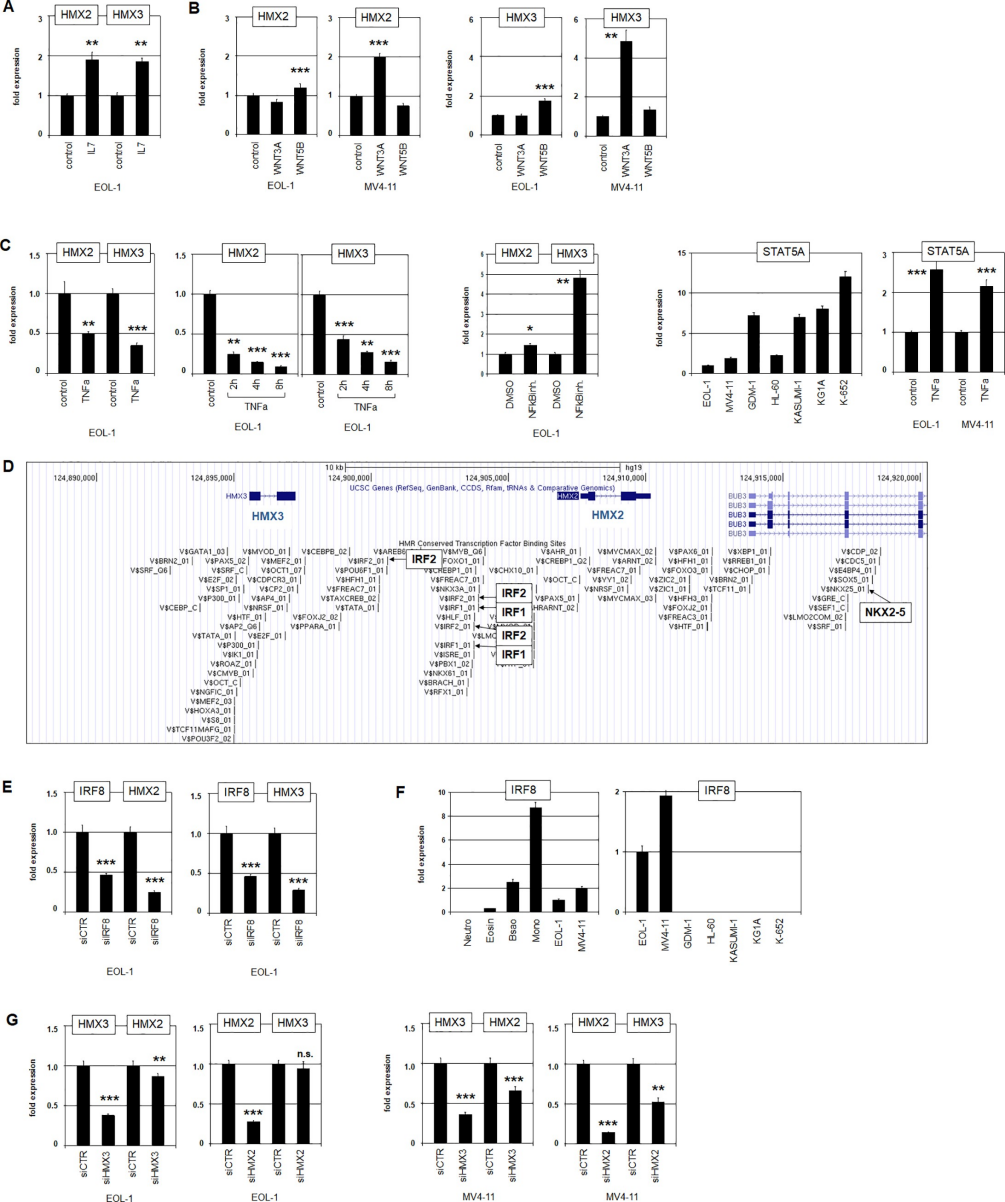
Identification of upstream factors regulating HMX2 and HMX3. (A) Treatment of EOL-1 cells with IL7 resulted in increased transcript levels of HMX2 and HMX3 as analyzed by RQ-PCR. Asterisks indicate calculated p-values obtained by t-Test analysis of medium controls and interleukin stimulation. (B) Treatment of EOL-1 and MV4-11 cells with WNT3A and WNT5B resulted in increased expression levels of HMX2 (left) and HMX3 (right). (C) Treatment of EOL-1 cells with NFkB-activator TNFa resulted in reduced expression levels of HMX2 and HMX3 (left). Treatment of EOL-1 cells with NFkB-inhibitor resulted in increased expression of HMX2 and HMX3 (middle). STAT5A served as marker for NFkB-activity which showed low expression levels and increased activity after stimulation with TNFa (right). (D) A genomic map of the locus for HMX2/3 was obtained from the UCSC genome browser, showing potential transcription factor binding sites including those for IRF1, IRF2 and NKX2-5. (E) RQ-PCR analysis of EOL-1 cells treated for siRNA-mediated knockdown of IRF8 resulted in elevated expression levels of HMX2 (left) and HMX3 (right), indicating an activating impact of IRF8. (F) Quantification of IRF8 by RQ-PCR in primary granulocytes and monocytes (left) and selected AML cell lines (right) demonstrated significant levels in EOL-1 and MV4-11. (G) RQ-PCR analysis of EOL-1 (left) and MV4-11 (right) treated for siRNA-mediated knockdown of HMX3 and HMX2 demonstrated slightly reduced expression levels of HMX2 and HMX3, respectively, indicating mutual activation.

Treatments of EOL-1 and MV4-11 cells with IL7, NFkB-activator TNFa, NFkB-inhibitor, WNT3A and WNT5B impacted expression of both genes (**Fig 3A–3C**), specifically showing that IL7- and WNT-signalling activated, and TNFa/NFkB-signalling inhibited expression of HMX2/3. WNT-signalling showed differences in HMX2/3 expression both with respect to the cell line and to the ligand (**Fig 3B**), implying that MV4-11 was more sensitive and WNT3A more effective. STAT5A expression can serve as a marker for NFkB-activity, of which it is a direct target gene [38, 39]. This gene, weakly expressed in EOL-1 and MV4-11, recovered after TNFa-treatment (**Fig 3C**), evidencing low NFkB-activity in these cell lines. A time-scale analysis showed fast and progressive inhibition of HMX2 and HMX3 by TNFa/NFkB within hours (**Fig 3C**), highlighting the suppressive impact of this pathway. Finally, RQ-PCR analysis confirmed elevated expression levels of IL7R in EOL-1 (**S8A Fig**). Additional sequence analysis of the transmembrane-encoding part of IL7R revealed an activating mutation in one EOL-1 allele, namely T245I (**S8A Fig**). These data supported oncogenic activity of this pathway in EOL-1.

In accordance with our expression profiling data, analysis of potential transcription factor binding sites at HMX2 and HMX3 showed several IRF-sites in their intergenic region (**Fig 3D**), suggesting a direct regulatory role for prominent IRF8 expression. Consistent with such a model, siRNA-mediated knockdown of IRF8 resulted in reduced transcription of HMX2 and HMX3, demonstrating an activating impact for this transcription factor on both genes (**Fig 3E**). Expression levels of IRF8 in EOL-1 and MV4-11 resembled that of primary basophils and lay above that of primary eosinophils (**Fig 3F**), confirming aberrant IRF8 activity in HMX2/3-positive cell lines. In addition to potential IRF-binding sites, the HMX2/3 locus also contains a site for another NKL homeobox gene, NKX2-5 (**Fig 3D**). Of note, the reported binding-sites for NKX2-5 and HMX factors are identical [40]. Therefore, we assumed a potentially mutual regulation of HMX2 and HMX3 which was examined by siRNA-mediated knockdown of both genes (**Fig 3G**). The results confirmed mutual activation which was more prominent for HMX3 in MV4-11.

Taken together, overexpressed IRF8, mutual regulation of HMX2 and HMX3, mutated IL7R and specific WNT-signalling mediated activation while TNFa/NFkB- signalling mediated inhibition of HMX2/3 expression. In addition, downregulated activity of this repressive pathway in HMX2/3-positive cell lines further enhanced the transcription of both NKL homeobox genes.

### Non-coding regulatory mutations mediate transcription of HMX2 and HMX3

**Fig 4:**
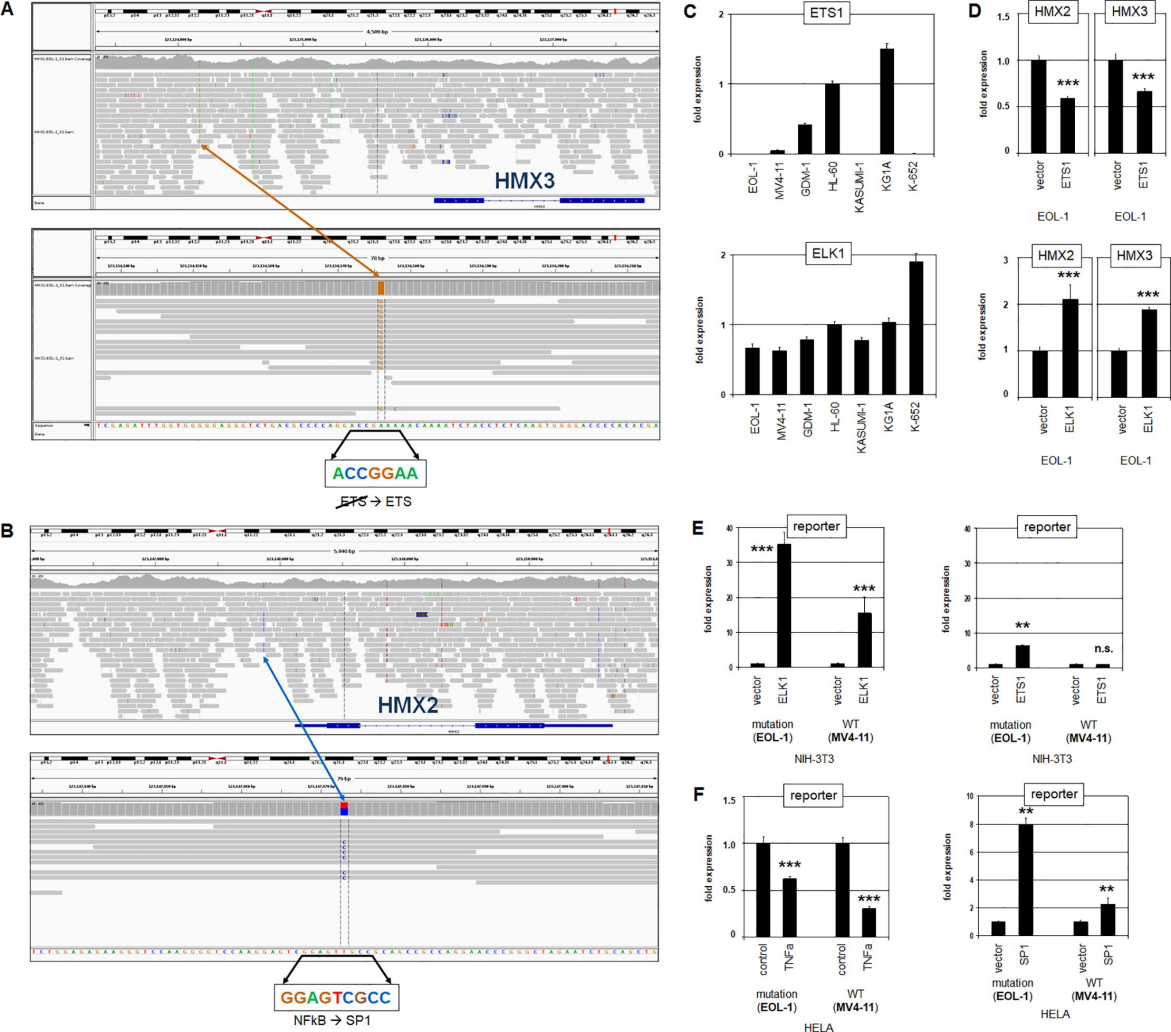
Regulatory mutations at HMX2/3 in EOL-1. The illustration of RNA-seq data from EOL-1 shows two different mutations at the HMX2/3 locus. (A) An A-to-G mutation in the 5´-region of HMX3 generates a consensus ETS-site. (B) A T-to-C mutation in the 5´-region of HMX2 transforms an NFkB-site into a SP1-site. (C) Quantification of ETS1 (above) and ELK1 (below) in selected AML cell lines by RQ-PCR. (D) Forced expression of ETS1 (above) and ELK1 (below) in EOL-1 cells resulted in respectively reduced and increased expression levels of both HMX2 and HMX3. Asterisks indicate calculated p-values obtained by t-Test analysis of controls (vector) and factor expression. (E) Reporter-gene assay in NIH-3T3 cells using a fragment containing a normal (obtained from MV4-11) or mutated (obtained from EOL-1) ETS-site. Forced expression of ELK1 (left) and ETS1 (right) resulted in elevated reporter-gene activity which was higher using ELK1 for the mutated fragment. (F) Reporter-gene assay in HELA cells using a fragment containing a normal (MV4-11) or mutated (EOL-1) NFkB-site. Treatment with NFkB-activator TNFa (left) resulted in reduced reporter-gene activity which was more pronounced using the normal fragment. Forced expression of SP1 (right) resulted in elevated reporter-gene activity which was higher using the mutated fragment.

Mutations of particular transcription factor binding sites reportedly effect deregulation of oncogenes and tumor suppressor genes in T-cell leukemia and AML, respectively [41, 42]. To investigate if this mechanism plays a role in HMX2/3 activation we performed whole genome sequencing of EOL-1, focussing our screen on the promoter and intergenic regions. The genomic sequence data of the HMX2/3 locus revealed two mutations, respectively altering two different potential transcription factor binding sites in this cell line. First, in the upstream region of HMX3 we identified an A-to-G mutation, generating of a novel consensus ETS-site: ACCGGAA (**Fig 4A**). Second, within the intergenic region upstream of HMX2 we identified a T-to-C mutation which transforms a potential NFkB-site into a non-consensus SP1-site: GGAGTCGCC (**Fig 4B**). Interestingly, both alterations chimed with our previous findings, showing that ETS1 levels were reduced (**S7B Fig**) and that NFkB-activator TNFa inhibited HMX2/3 expression (**Fig 3C**).

RQ-PCR analysis confirmed absent expression of ETS1 in EOL-1 and MV4-11 while ETS-factor ELK1 was rather uniformly expressed in AML cell lines (**Fig 4C**). Consistent with this picture, forced expression of ETS1 in EOL-1 inhibited expression of both HMX2 and HMX3 while ELK1 boosted their activity (**Fig 4D**). ChIP-seq data for ELK1 generated in HELA-S3 cells showed strong binding at the transcription start site of HMX3 and weak binding in the upstream region matching the position of the detected mutation in EOL-1 (**S9 Fig**) [43]. These data may indicate that the binding of ELK1 is enhanced by this mutation. Thus, specific ETS-factors contrastingly regulated HMX2/3 transcription (via this novel ETS-site).

To scrutinize these mutations in more detail we performed reporter-gene assays for normal and mutated regulatory genomic fragments cloned in front of a reporter gene. Accordingly, we transfected NIH-3T3 cells with reporter-constructs containing either an incomplete or the novel consensus ETS-site. Additionally, the cells were transfected with an expression-construct for ETS-factor ELK1 (**Fig 4E**). Our data demonstrated that ELK1 activated the mutated construct significantly more strongly than the wild type sequence. Additional transfection of an expression-construct for ETS1 also activated the mutated construct more strongly, however, this effect was much weaker as compared to ELK1 (**Fig 4E**). These data show that both, ETS1 and ELK1 are activators of HMX2/3. However, the activating potential of ELK1 is stronger as compared to ETS1. ETS1 and ELK1 may compete for binding at HMX3. Therefore, overexpressed ETS1 replaced ELK1 which resulted in reduced activation. Together, this mutation enhanced the activating effects of ETS-factors.

Next, TNFa-sensitive HELA cells were transfected with reporter-constructs containing normal or mutated NFkB-sites and additionally treated with NFkB-activator TNFa (**Fig 4F**). The results showed that TNFa inhibited the reporter-gene significantly more via the normal site, suggesting that this mutation reduced binding of suppressive NFkB. Furthermore, forced expression of SP1 resulted in elevated reporter-gene activity which was significantly higher using the construct with the mutated site (**Fig 4F**). Thus, this mutation decreased the inhibitory input of NFkB and simultaneously increased activation by the general transcription factor SP1.

Taken together, this strategy unmasked two mutated transcription factor binding sites in AML cell line EOL-1 which modulated aberrant HMX2/3 expression via altered effects of ETS1 and ELK1, and of NFkB and SP1.

### HMX2 inhibits eosinophilic differentiation in AML

**Fig 5:**
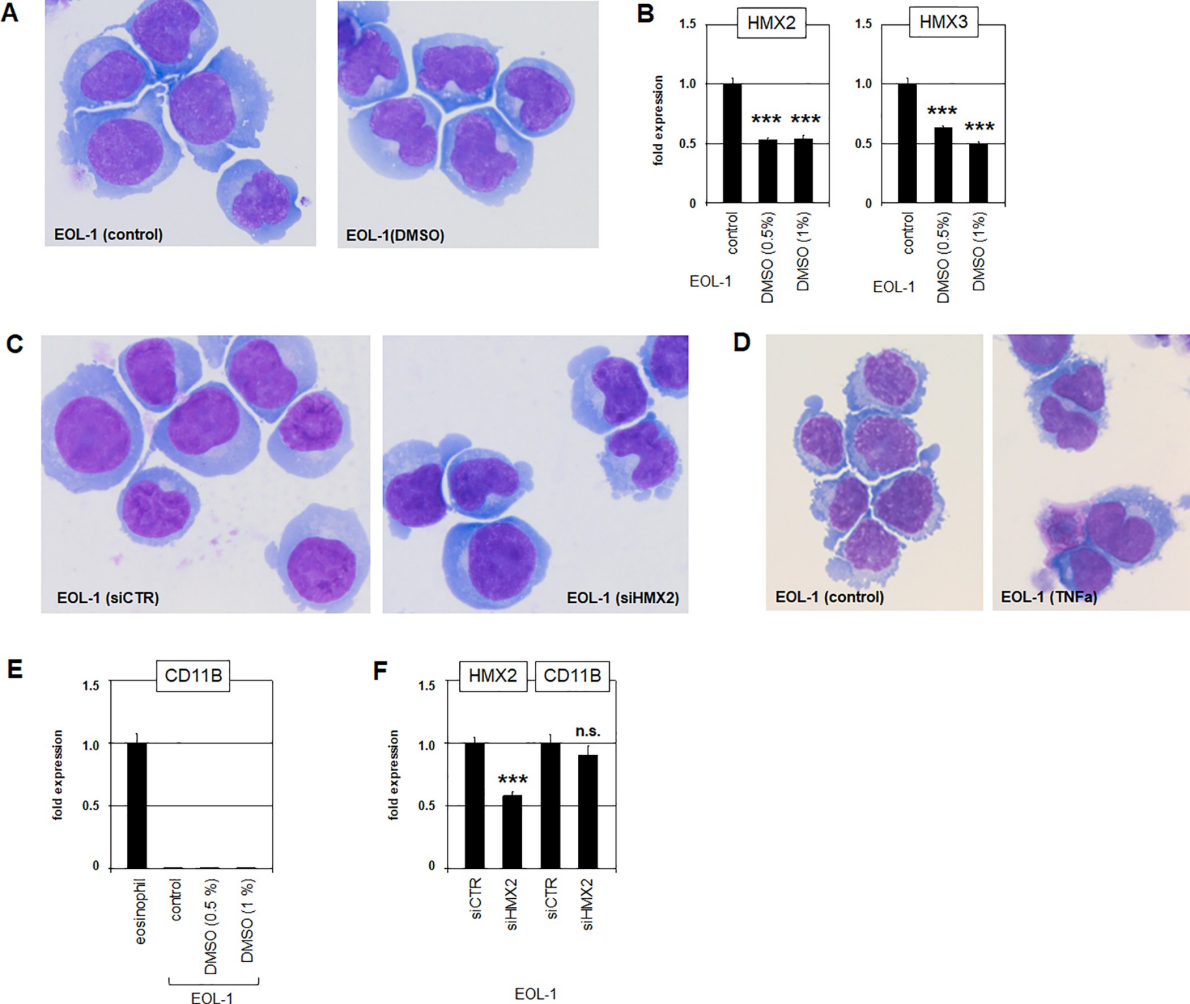
Differentiation of EOL-1. (A) Treatment of EOL-1 cells with DMSO for three days induced morphological alterations as shown by Giemsa-May-Grünwald staining. The nuclei became typically indented like kidney beans which can be interpreted as eosinophilic differentiation. (B) The same treatment resulted in decreased expression of HMX2 (left) and HMX3 (right) as analyzed by RQ-PCR. SiRNA-mediated knockdown of HMX2 (C) or treatment with TNFa (D) generated the same nuclear indentation as visible after DMSO-treatment, indicating that HMX2/3 inhibited eosinophilic differentiation. (E) Marker expression. (F) Marker expression. Asterisks indicate calculated p-values obtained by t-Test analysis of controls (siCTR) and siRNA-targeted knockdown.

EOL-1 aberrantly expressed HMX2 and HMX3 and has been shown to differentiate into eosinophilic cells after DMSO-treatment [44]. Therefore, this cell line may represent a suitable model to investigate the role of deregulated NKL homeobox genes in differentiation arrest. EOL-1 cells treated with 1% DMSO for three days underwent morphological changes indicative of resumed eosinophilic cell differentiation (**Fig 5A**). Furthermore, this treatment reduced expression levels of HMX2 and HMX3 (**Fig 5B**), implicating collusive HMX2/3 activity and cell differentiation. Interestingly, siRNA-mediated knockdown of HMX2 or treatment with HMX2/3-inhibitor TNFa induced similar alterations of the cell morphology (**Fig 5C and 5D**), showing a causal connection between HMX2/3 activity and eosinophilic differentiation arrest. An established marker for eosinophilic cell differentiation is CD11B [45]. However, its expression level did not raise after DMSO treatment or after siRNA-mediated knockdown of HMX2 (**Fig 5E and 5F**), indicating incomplete cell differentiation. Together, these experiments documented that HMX2/3 are involved in the differentiation arrest which is pathognomonic for leukemic transformation.

### Downstream activities of HMX2 and HMX3 in EOL-1

**Fig 6:**
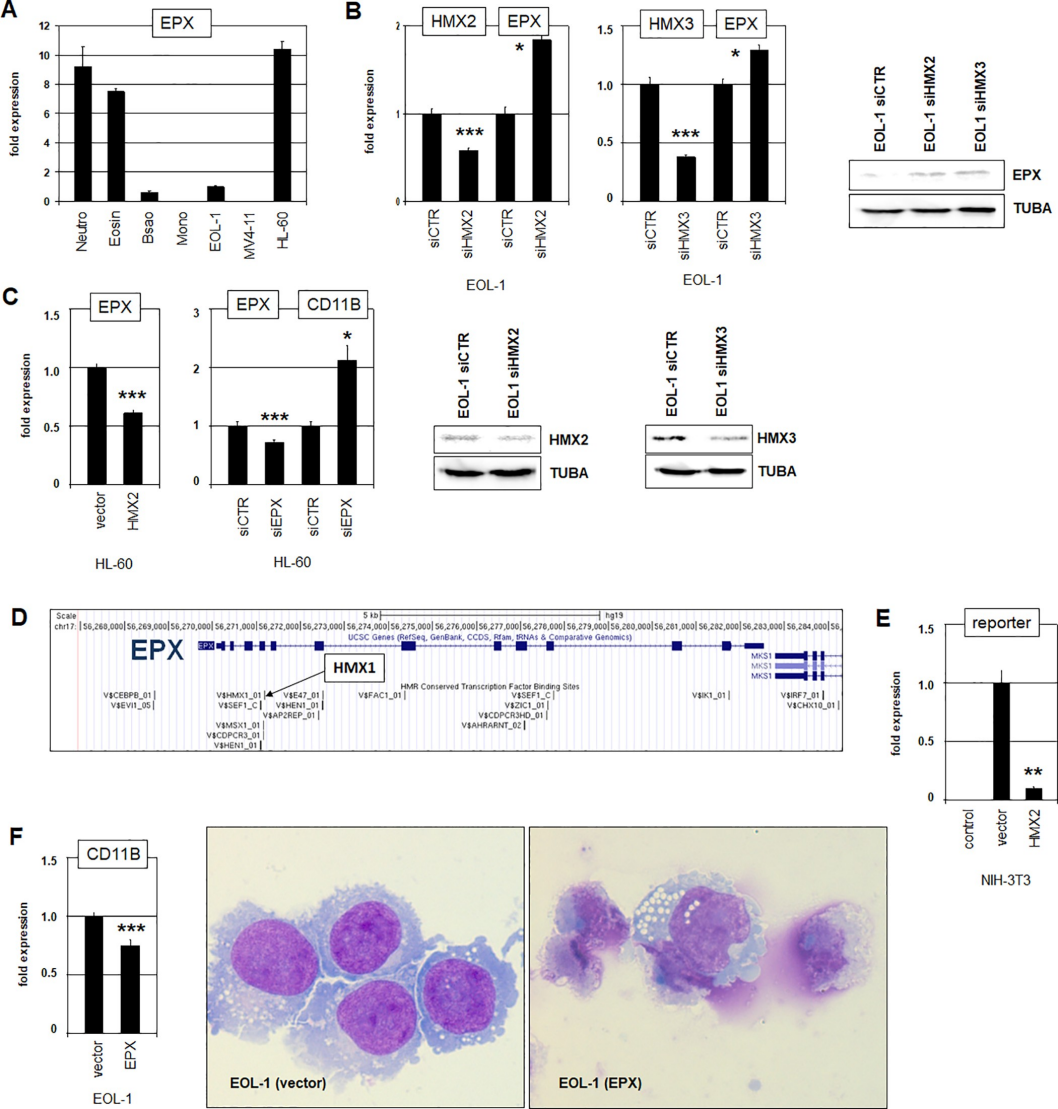
Regulation of EPX by HMX2 and HMX3. (A) Quantification of EPX expression in primary granulocytes (neutrophils, eosinophils, basophils) and selected cell lines by RQ-PCR. (B) SiRNA-mediated knockdown of HMX2 (left) and HMX3 (right) resulted in elevated expression levels of EPX. Reduced expression of HMX2 and HMX3 and elevated expression of EPX was also shown by Western blot analysis. Asterisks indicate calculated p-values obtained by t-Test analysis of controls (siCTR) and siRNA-targeted knockdown. (C) Forced expression of HMX2 in HL-60 cells resulted in reduced expression level of EPX (left). SiRNA-mediated knockdown of EPX resulted in activation of CD11B expression (right). (D) A genomic map of the locus for EPX was obtained from the UCSC genome browser, showing potential transcription factor binding sites including one for HMX1. (E) Reporter-gene assay in NIH-3T3 cells using a fragment containing the identified HMX-site. Forced expression of HMX2 resulted in decreased reporter-gene activity, demonstrating a direct repressive impact. (F) Forced expression of EPX in EOL-1 slightly reduced the expression level of CD11B. Microscopical inspection of Giemsa-May-Grünwald-stained EOL-1 cells indicated strongly induced apoptosis by forced EPX expression as possible consequence of continued differentiation. Living cells resemble differentiated cells after DMSO-induction.

To understand the mechanism(s) of eosinophilic differentiation arrest it may be helpful to identify target genes of the oncogenic NKL homeodomain factors HMX2 and HMX3 in AML cells. EPX encodes eosinophil peroxidase fundamental for both, function and development of eosinophils [46]. RQ-PCR analysis showed high EPX expression levels in primary eosinophils and neutrophils in addition to cell line HL-60 but reduced levels in EOL-1 and MV4-11 (**Fig 6A**), highlighting the proposed functional relation between expression levels and cell differentiation in eosinophilic AML. SiRNA-mediated knockdown of HMX2 and HMX3 in EOL-1 cells resulted in elevated expression of EPX (**Fig 6B**), while forced expression of HMX2 in HL-60 cells proved inhibitory (**Fig 6C**). However, knockdown experiments in HL-60 indicated an inhibitory effect of EPX on CD11B expression (**Fig 6C**). Transcription factor binding site analysis indicated an HMX-site in the 5´-part of EPX implying direct interaction (**Fig 6D**). Accordingly, we performed a reporter-gene assay for this site. The results demonstrated reduced reporter-gene activity after forced HMX2 expression, indicating direct suppression of EPX by HMX2 (**Fig 6E**). Forced expression of EPX in EOL-1 cells rather inhibited expression of differentiation marker CD11B (**Fig 6F**). But microscopical inspection indicated that EPX drives the cells into apoptosis as possible consequence of continued differentiation (**Fig 6F**). Therefore, these data suggest that EPX plays an important role in aberrant differentiation arrest of EOL-1 cells.

Comparative expression profiling data additionally revealed potential HMX target genes in EOL-1 including the selected candidates PDGFRA, DLX1/2, and HTR7 (**S1 Table, S7A Fig**) which were analyzed in the following. In EOL-1, PDGFRA is fused to FIP1L1 by a small genomic deletion (**S6A and S6B Fig**) [36]. This aberration is accompanied by reduced expression levels of widely expressed FIP1L1 and ectopic activation of PDGFRA (**S6C Fig**). Interestingly, the FIP1L1 gene contains a binding site for NKL homeodomain factor NKX2-5 (**S6D Fig**), which may represent a site for HMX-factors [40], suggesting an activatory impact of HMX2/3 on the FIP1L1-PDGFRA fusion gene. Accordingly, siRNA-mediated knockdown of HMX2 resulted in reduced expression levels of PDGFRA, supporting this potential interaction (**S6E Fig**). Interestingly, HMX3 showed no impact in PDGFRA expression (**S6E Fig**), indicating functional differences between these closely related transcription factors.

**Fig 7:**
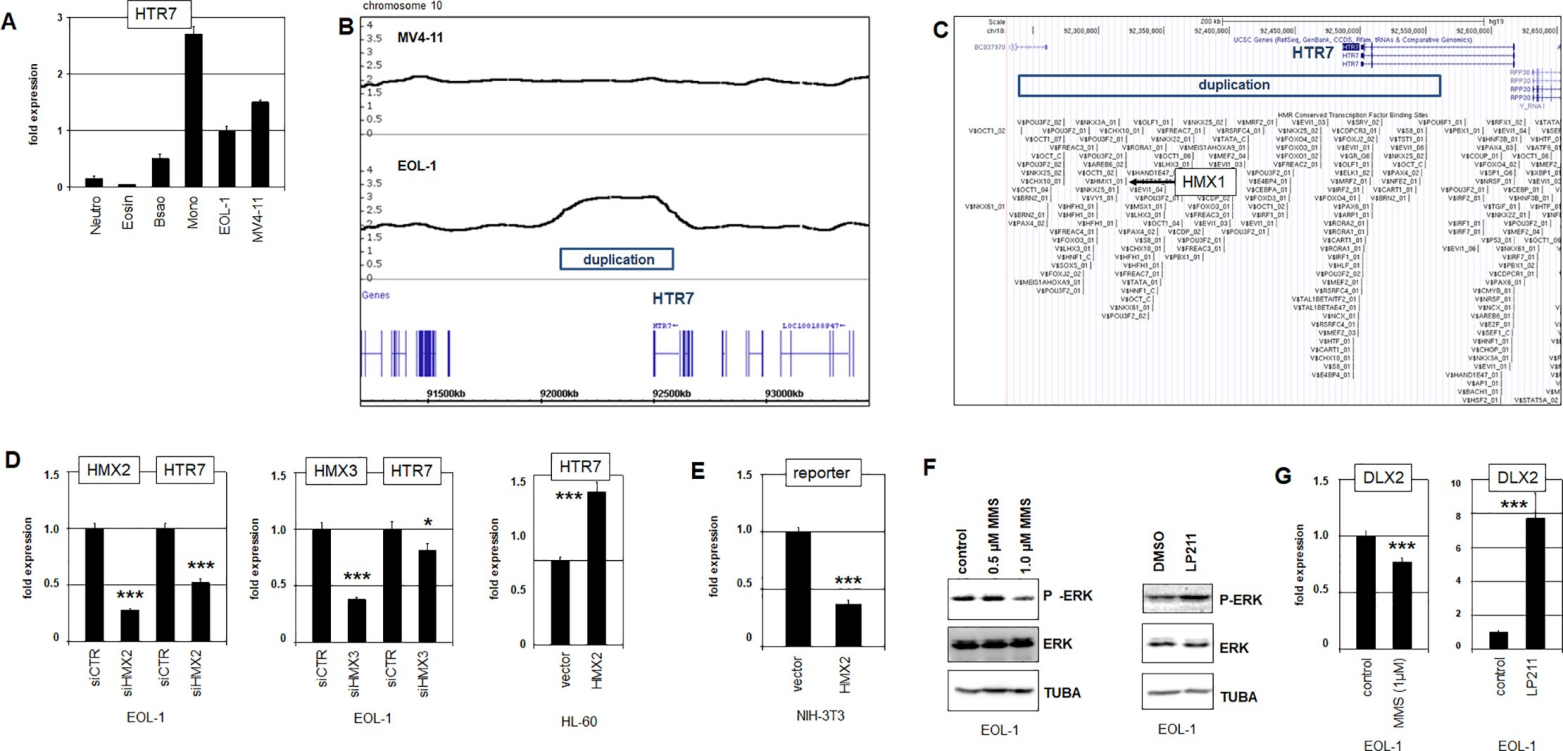
Regulation of HTR7 by HMX2 and HMX3. (A) Quantification of HTR7 expression in primary granulocytes (neutrophils, eosinophils, basophils), monocytes, and selected cell lines by RQ-PCR. (B) Genomic profiling data demonstrated a duplication in EOL-1 which encompassed the flanking regulatory region of HTR7. (C) A genomic map of the locus for HTR7 was taken from the UCSC genome browser, showing potential transcription factor binding sites. The indicated duplication detected in EOL-1 containes a potential HMX1-site. (D) SiRNA-mediated knockdown of HMX2 (left) and HMX3 (middle) resulted in reduced expression levels of HTR7, indicating an activating impact. Asterisks indicate calculated p-values obtained by t-Test analysis of controls (siCTR) and siRNA-targeted knockdown. Forced expression of HMX2 in HL-60 resulted in elevated transcript levels of HTR7 (right), supporting an activating impact. (E) Reporter-gene assay in NIH-3T3 cells using a fragment containing the identified HMX1-site. Forced expression of HMX2 resulted in decreased reporter-gene activity, demonstrating a direct regulatory impact. (F) Treatment of EOL-1 cells with HTR7-inhibitor MMS and HTR7-activator LP211 resulted in decreased and increased levels of ERK-phosphorylation, respectively, as shown by Western blot. (G) Treatment of EOL-1 cells with HTR7-inhibitor MMS and HTR7-activator LP211 resulted in decreased and increased levels of DLX2-transcription, respectively, as shown by RQ-PCR.

DLX1 and DLX2 belong to the NKL homeobox gene subclass as well and are aberrantly activated in AML via FLT3/ERK-signalling [19]. Likewise, EOL-1 contained an activated ERK-pathway and expressed DLX1 and DLX2 (**Fig 2E, S8B Fig**). HTR7 encodes the 5-hydroxytryptamine receptor 7 which activates the ERK-pathway [47]. RQ-PCR analysis demonstrated high expression levels of HTR7 in primary monocytes, low in primary eosinophils, and raised in EOL-1 and MV4-11 cells (**Fig 7A**), indicating aberrant activity in eosinophilic cell line EOL-1. Our profiling data showed for EOL-1 a genomic gain of the regulatory downstream region of HTR7 (**Fig 7B**). Moreover, this region contains a potential binding site for HMX transcription factors (**Fig 7C**), suggesting a direct regulatory role. SiRNA-mediated knockdown of HMX2 or HMX3 resulted in reduced expression levels of HTR7 (**Fig 7D**), demonstrating that both factors activate HTR7 in EOL-1 cells. Accordingly, forced expression of HMX2 in HL-60 mediated activation of HTR7 transcription (**Fig 7D**). In addition, we performed a reporter-gene assay for this site in NIH-3T3 cells. Unexpectedly, HMX2 inhibited the reporter gene activity while confirming direct regulation of HTR7 by HMX2 via this site (**Fig 7E**). Functional analysis of HTR7 was performed by treatment of EOL-1 with HTR7-inhibitor MMS and HTR7-activator LP211. These stimulations resulted in suppressed and enhanced ERK-activity, respectively, as indicated by Western blot analysis of phospho-ERK (**Fig 7F**). As expected, treatments with MMS and LP211 caused respectively suppressed and enhanced expression of ERK-target gene DLX2 (**Fig 7G**). Thus, HTR7 is a direct target gene of HMX2 and HMX3 and contributes to aberrant activation of DLX2 via the ERK-pathway. Of note, since HMX2/3 are negatively regulated by ERK-signalling, HTR7 activation forms a negative feedback loop together with these NKL homeobox genes.

Taken together, we identified HMX2/3 mediated suppression of eosinophilic differentiation factor EPX, HMX2 supported expression of fusion-gene FIP1L1-PDGFRA, and HMX2/3 mediated activation of receptor gene HTR7. These deregulated target genes contribute to arrested cell differentation and activate the oncogenic ERK-pathway, highlighting the leukemic potential of HMX2 and HMX3.

## Discussion

**Fig 8:**
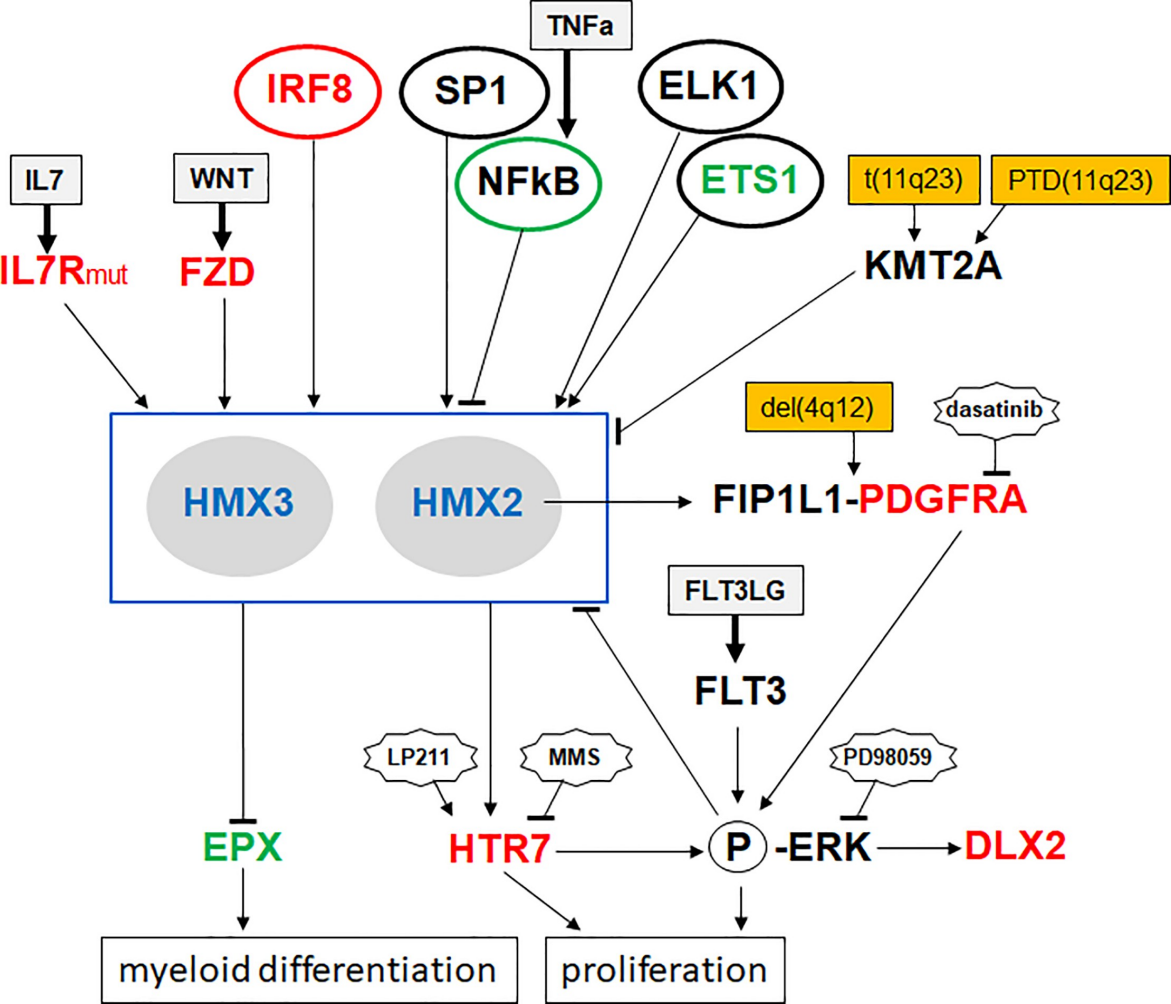
Gene regulatory network for HMX2 and HMX3 in AML. This diagram summarizes the results of this study, showing a gene regulatory network. HMX2 and HMX3 are located centrally, activating and repressing factors and pathways are indicated above, and the target genes EPX and HTR7 below. Deregulation of EPX and HTR7 indicates aberrant effects for differentiation and proliferation. Genomic rearrangements targeting KMT2A or PDGFRA are indicated.

According to the reported myeloid NKL-code, six NKL homeobox genes participate in the physiological differentiation of granulocytes (basophils, eosinophils, neutrophils), monocytes and macrophages, dendritic cells, and erythrocytes while 24 deregulated members of the NKL subclass are described in AML and MDS [18]. Here, we analyzed the role of the NKL homeobox genes HMX1, HMX2 and HMX3 in AML. In this trio HMX1 stands somewhat alone as it is normally expressed in erythropoiesis and aberrantly activated in MDS but not in AML, as shown previously and supported in this study [18]. In AML we demonstrated aberrant expression of HMX2 and HMX3, indicating functional differences with the closely related HMX1. The observed coexpression of HMX2 and HMX3 may reflect their genomic organization as direct neighbors at chromosomal position 10q26 while HMX1 is located at 4p16 and, therefore, not associated with another HMX-group member. In this study, we uncovered several upstream and downstream genes and pathways for HMX2 and HMX3 activity which conspire to form an aberrant gene regulatory network. A summary of this network is depicted in **Fig 8** and further discussed below.

Expression profiling data indicated that deregulation of HMX2, normally rare in AML patients, is much higher in those with KMT2A-rearrangements. Using cell lines as models, we excluded straightforward mutual activation of KMT2A and HMX2/3. Accordingly, AML cell lines ML-2, MONO-MAC-6, MUTZ-11 and NOMO-1 express KMT2A-fusion genes while HMX2 and HMX3 were silent, also demonstrating absence of an activatory input of these KMT2A-aberrations [18]. Thus, although HMX2/3 expression and KMT2A rearrangements showed a strong correlation, we were unable to uncover a direct regulatory connection. In addition, KMT2A does not influence the expression of deregulated NKL homeobox gene NANOG in AML cell line NOMO-1 [18] which may support the view that H3K4-methylation is probably not involved in leukemic activation of this group of homeobox genes. Interestingly, a case report on a child with intellectual disability showed both KMT2A-deletion and eosinophilia [48], indicating tumor suppressor gene activity for KMT2A in this malignancy. Therefore, we speculate that NKL homeobox genes HMX2/3 and rearranged KMT2A operate synergistically to promote myeloid leukemogenesis.

Our data in AML cell lines revealed an activating impact of ETS1 and ELK1 in HMX2/3 expression, indicating oncogenic potential of ETS-factors in this context. This finding is supported by two reports about the relation between KMT2A and ETS1 in AML/MDS: both genes are coamplified and overexpressed [49], and the fusion KMT2A-EB1 (but not KMT2A-AF10) activates the expression of ETS1 [50]. These results indicate that ETS1 operates as an oncogene in the context of rearranged KMT2A in AML. Thus, overexpressed ETS1 may also support the oncogenic activity of NKL homeobox genes HMX2/3.

In search of upstream factors and pathways regulating the expression of HMX2/3 in AML we identified activatory (IL7, WNT) and inhibitory (ERK, NFkB) pathways. Activating IL7R mutations as identified here in EOL-1 are mostly located in the transmembrane domain. These mutations generate either a cystein or a hydrophobic amino acid residue in this protein region which enhance dimerization and subsequent activation of the receptor molecules [51].

Furthermore, we identified IRF8 as activating transcription factor for HMX2/3. The intergenic region of HMX2 and HMX3 contains several IRF binding sites, indicating direct regulation by this factor. Normally, IRF8 regulates the development of myeloid cells. It promotes the formation of monocytes, basophils and eosinophils and inhibits the differentiation of neutrophils [52]. In eosinophilic cell line EOL-1 we detected enhanced IRF8 levels as compared to normal eosinophils, suggesting aberrant activation which may interfere with eosinophilic differentiation and contribute to HMX2/3 expression.

Our genomic profiling data showed a small deletion at 3p13 targeting the 3´-part of FOXP1 which may underlie reduced expression levels in EOL-1 (**S5 Fig, S8C and S8D Fig**), indicating tumor suppressor activity for this gene. Expression profiling analysis of normal myeloid cells from the peripheral blood demonstrated elevated FOXP1 expression in eosinophils and basophils while neutrophils, monocytes and mast cells showed significantly lower levels (**S8E Fig**). These data indicated that FOXP1 represents a myeloid transcription factor involved in eosinophilic and basophilic differentiation. Eosinophilia-specific PDGFRA-fusion with FOXP1 disrupts this gene [53], supporting tumor suppressor activity in this malignancy. Furthermore, FOXP1 is a physiological target gene of HMX1 in the retina [54]. Accordingly, FOXP1 contains a potential HMX binding site which may mediate regulation by HMX1 as well as HMX2/3 (**S8F Fig**). Thus, in the malignant context, FOXP1 is disrupted by translocation or deletion which results in reduced expression levels and consequently disturbed differentiation. In EOL-1 cells deletion of FOXP1 may additionally prevent its potential activation by HMX2/3.

NKL homeobox genes encode basic developmental regulators and show tissue specific expression patterns in embryonal development. Mutations or loss of NKL homeobox gene expression is associated with corresponding developmental defects: aberrations of HMX1 result in malformation of the outer ear and those of HMX2/3 in inner ear defects and deafness [55, 56]. Expression of HMX3 in the otic epithelium begins first, otherwise HMX2 and HMX3 are coexpressed in the innner ear [32]. Knockouts for HMX2 [57], HMX3 [58], or both genes [32] show functional redundancy of the genes which may reflect their evolutionary history and sequence similarity. Conserved gene clustering combined with coexpression as observed for HMX2/3 may reflect shared regulatory sites. Accordingly, the locus of HMX2/3 containes several conserved non-coding elements, indicating that these parts are involved in evolutionary conserved regulations of their gene activity [59, 60]. This type of tandem arrangement is also present at the loci for NKL homeobox genes of the DLX family including DLX1 and DLX2. Moreover, DLX1/2 are coexpressed in EOL-1 and also share regulatory elements in their intergenic regions [61]. Interestingly, DLX1/2 are in addition to DLX3 and DLX5/6 involved in early development of the inner ear, coinciding with HMX2/3 [22, 62].

Furthermore, we identified two mutations in transcription factor binding sites, revealing impacts for ETS1, ELK1, NFkB and SP1 in HMX2/3 regulation. Mutations of particular transcription factor binding sites play also an oncogenic role in T-cell leukemia and AML [41, 42]. The incidence of such mutations in cancer is low which may reflect difficulties in identification and functional analysis. Moreover, many transcription factors are able to interact with differing binding sites which may lead to the low penetrance of some mutations. However, a recent study of 2.658 whole cancer genomes shows that mutations in non-coding genes and regulatory sequences are indeed less frequent than mutations in protein-coding genes [63]. Nevertheless, this type of mutation may represent an important fraction of malignant alterations providing diagnostic and/or prognostic information, thus, deserving closer examination.

The myeloid NKL-code reflects correlations between NKL homeobox gene activities and particular premature and terminal myeloid differentiation stages [18]. Accordingly, aberrant activities of NKL homeobox genes may disturb these processes by deregulation of specific target genes, resulting in altered development or in differentiation arrest. Therefore, oncogenic target genes of aberrantly expressed HMX2 and HMX3 should contain their binding sites and encode disease-relevant proteins. DNA-binding sites for HMX2 and HMX3 are identical although slight differences exist [64]. Furthermore, these binding sites overlap NKX2-5 [40], indicating shared target gene regulation of these NKL subclass members. Here, we revealed that HMX2 but not HMX3 mediated activation of the fusion-oncogene FIP1L1-PDGFRA probably via an NKX2-5 site, showing functional differences between these similar proteins. The observation that aberrantly expressed NKL homeobox genes activate genomically rearranged oncogenes has been also reported in T-ALL where NKX3-2 contributes to the expression of translocated NKX2-5 [65].

In addition, we identified in this study two aberrantly regulated target genes of HMX2/3, namely EPX and HTR7. The former is a marker gene for myeloid differentiation [66]. More importantly, EPX expression is required for eosinophilic differentiation [46], highlighting our finding that HMX2/3 suppress both, EPX activity and cell differentiation. HTR7 enhanced the ERK-pathway, oncogenic in AML [19]. Thus, both identified HMX2/3 target genes represent powerful tumor suppressors and oncogenes, respectively, and their deregulation may critically promote leukemogenesis.

In hematopoietic cancers NKL homeobox genes are frequently overexpressed. This deregulation comprises both NKL-code members and NKL homeobox genes normally not expressed in hematopoiesis [18]. The data of this study support the view that this homeobox gene subclass substantially impacts developmental processes and cell differentiation in the myeloid context. Therefore, this group of oncogenes deserves more attention in the clinical diagnostic and may represent a novel therapeutic target in AML.

## Supporting information

S1 Fig Expression profiling data of AML patients and cell lines. (A) Dataset GSE15434 contains AML patients with normal kyryotype. (B) Dataset GSE61804 contains AML patients with normal/abnormal karyotype. (C) Dataset GSE21261 contains AML patients with MDS/nos. (D) Dataset GSE19577 contains AML patients with KMT2A rearrangements. (E) Dataset GSE59808 contains AML cell lines. Of note, analysis of this dataset revealed four HMX2-positive cell lines, namely EOL-1, MOLM-13, MV4-11 and SH-1. However, cell line SH-1 is confusing because there are described cell lines with similar names but SH-1 does not exist. Therefore, we excluded this cell line from further examinations.
(TIF)

S2 Fig Gene-annotation enrichment analysis of AML patients and cell lines. (A) Dataset GSE15434 contains AML patients with normal karyotype. (B) Dataset GSE61804 contains AML patients with normal/abnormal karyotype. (C) Dataset GSE21261 contains AML patients with MDS/nos. (D) Dataset GSE19577 contains AML patients with KMT2A rearrangements. (E) Dataset GSE59808 contains AML cell lines. We used for GAEA HMX2-positive cell lines EOL-1, MOLM-13 and MV4-11 but not SH-1.
(TIF)

S3 Fig Expression of HMX1, HMX2 and HMX3 in AML cell lines. According to RNA-seq data from LL-100 and setting the cut-off at 500, these data show absent expression of HMX1 in all cell lines while HMX2 and HMX3 are active in selective cell lines from different origin.
(TIF)

S4 Fig KMT2A-fusion genes in AML cell lines. (A) RT-PCR analysis of KMT2A-fusions in selected AML cell lines demonstrates KMT2A-AFF1 in MV4-11 (left) and KMT2A-MLLT3 in MOLM-13 (right). NTC: no template control. (B) RT-PCR analysis in selected AML cell lines of KMT2A (left) and of KMT2A-PTD (right). (C) LL-100 data for KMT2A RNA expression.
(TIF)

S5 Fig Genomic profiling data for EOL-1, MV4-11 and MOLM-13. Genomic profiling shows copy number alterations at 4q12 (FIP1L1-PDGFRA), 10q23 (HTR7), and 11q23 (KMT2A) in EOL-1. EOL-1 and MOLM-13 share a deletion at 9p21 containing CDKN2B. In MOLM-13, this deletion is involved in ins(11;9)(q22;p23) generating fusion gene KMT2A-MLLT3. No aberrations were found at the HMX2/3 locus at 10q26.
(TIF)

S6 Fig Fusion gene FIP1L1-PDGFRA. (A) Genomic profiling data show a deletion in EOL-1 at 4q12 which targets FIP1L1 and PDGFRA and removes CHIC2. (B) RT-PCR analysis of FIP1L1-PDGFRA (left) and of FIP1L1 (right) as control. (C) LL-100 data for FIP1L1, PDGFRA and CHIC2. (D) A genomic map of the locus for FIP1L1 was taken from the UCSC genome browser, showing potential transcription factor binding sites including a potential NKX2-5-site. (E) SiRNA-mediated knockdown of HMX2 (left) resulted in reduced expression levels of PDGFRA, indicating an activating impact while knockdown of HMX3 showed no alteration (right).
(TIF)

S7 Fig Comparative gene expression profiling analyses of cell lines. (A) Lists of differentially expressed genes in EOL-1 and MV4-11 as compared to the controls GDM-1, HL-60 and KG-1. Genes are arranged in the order of fold expression differences. (B) Gene-annotation enrichment analysis for AML cell lines EOL1 and MV4-11 using the top-1000 upregulated genes. Identified KEGG-pathways included JAK-STAT- and WNT-pathway. (C) Gene-annotation enrichment analysis for AML cell lines EOL1 and MV4-11 using the top-1000 downregulated genes. Identified KEGG-pathways included the NFkB-pathway.
(TIF)

S8 Fig Gene analyses. (A) RQ-PCR analysis of IL7R in selected AML cell lines (left). Sequencing results of cloned PCR products encompassing the TM-domain of IL7R (right). For MV4-11 we obtained five wildtype sequences, for EOL-1 we obtained three mutated and six wildtype sequences. (B) LL-100 data for DLX1 and DLX2 RNA expression. (C) Genomic profiling data show a deletion in EOL-1 at 3p13 which targets FOXP1. (D) LL-100 data for FOXP1 RNA expression. (E) FOXP1 expression data for primary cells obtained from dataset GSE109346. (F) A genomic map of the locus for FOXP1 was taken from the UCSC genome browser, showing potential transcription factor binding sites including a potential HMX1-site.
(TIF)

S9 Fig ChIP-seq data for ELK1 in HELA-S3 (ENCODE). The peaks at the transcriptional start site and in the upstream region (red arrow, corresponding to the mutated site in EOL-1) indicate ELK1 interaction at the HMX-locus.
(TIFF)

S1 Raw images Uncropped Western blots. (TIF)

S1 Table Comparative expression profiling data of selected AML cell lines. Group 1 (EOL-1 and MV4-11) has been compared to group 2 (GDM-1, HL-60 and KG1). Expressed genes are listed according their log-fold expression level. Additionally indicated are average expression levels and adjusted p-values. Upregulated and downregulated genes in group 1 are labeled in orange and green, respectively.
(XLSX)
