# Supplementary figures and images for: Aberrant expression of NKL homeobox genes HMX2 and HMX3 interferes with cell differentiation in acute myeloid leukemia
Source: PLoS One. 2020 Oct 13;15(10):e0240120. doi: 10.1371/journal.pone.0240120 (PMC7553312; doi:10.1371/journal.pone.0240120)

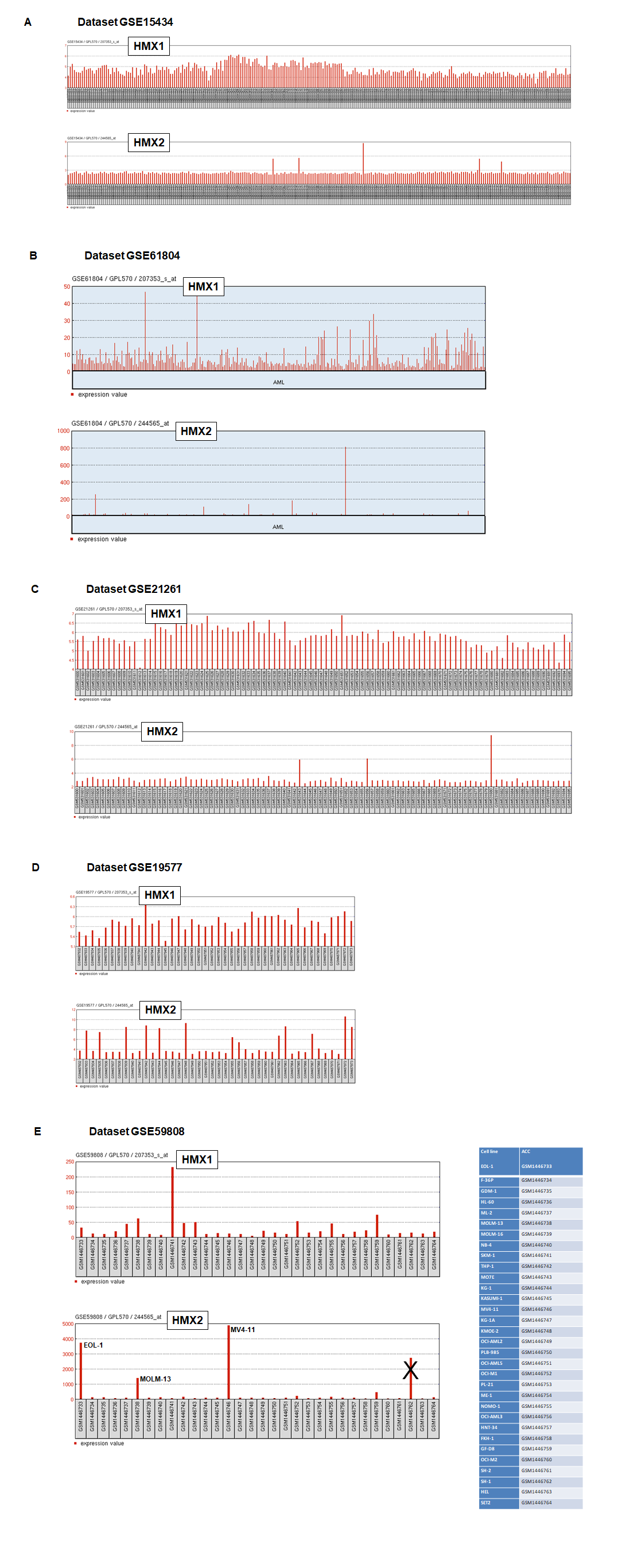

Supplement: S1 Fig — (A) Dataset GSE15434 contains AML patients with normal kyryotype. (B) Dataset GSE61804 contains AML patients with normal/abnormal karyotype. (C) Dataset GSE21261 contains AML patients with MDS/nos. (D) Dataset GSE19577 contains AML patients with KMT2A rearrangements. (E) Dataset GSE59808 contains AML cell lines. Of note, analysis of this dataset revealed four HMX2-positive cell lines, namely EOL-1, MOLM-13, MV4-11 and SH-1. However, cell line SH-1 is confusing because there are described cell lines with similar names but SH-1 does not exist. Therefore, we excluded this cell line from further examinations. (TIF) [file pone.0240120.s001.tif]

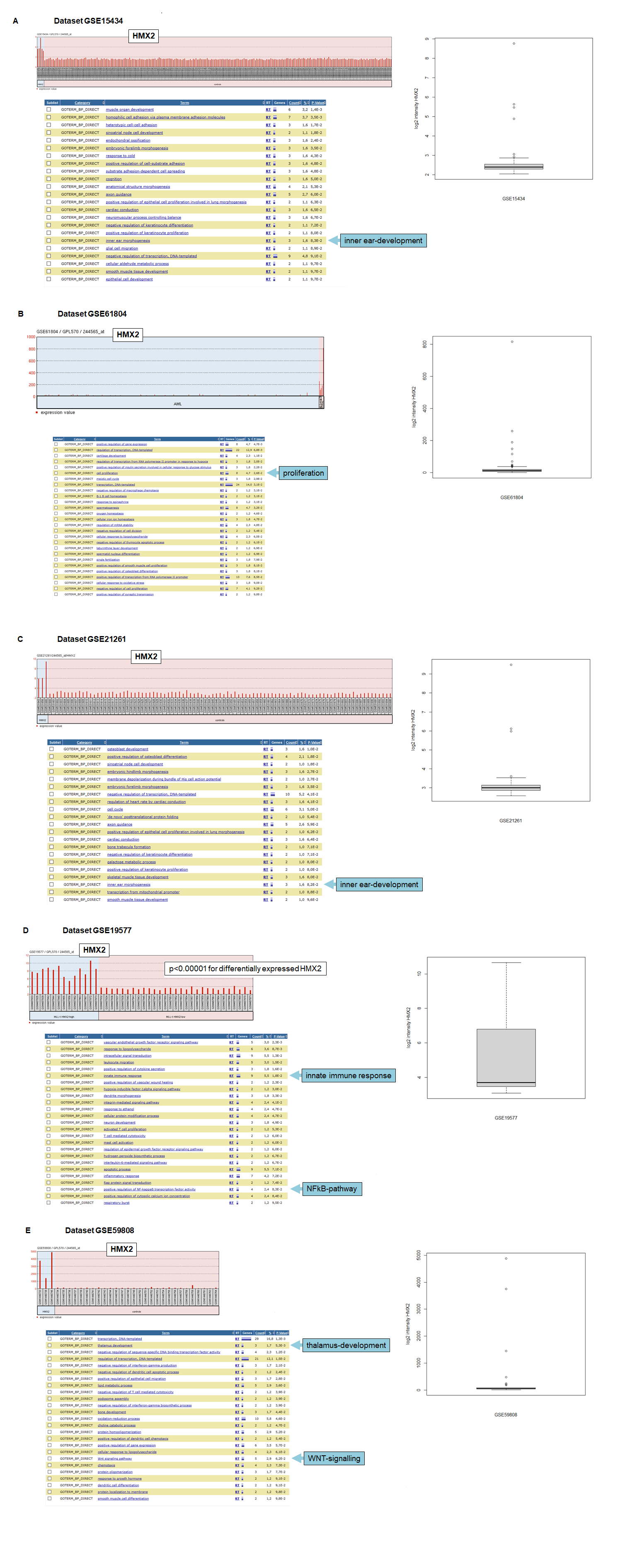

Supplement: S2 Fig — (A) Dataset GSE15434 contains AML patients with normal karyotype. (B) Dataset GSE61804 contains AML patients with normal/abnormal karyotype. (C) Dataset GSE21261 contains AML patients with MDS/nos. (D) Dataset GSE19577 contains AML patients with KMT2A rearrangements. (E) Dataset GSE59808 contains AML cell lines. We used for GAEA HMX2-positive cell lines EOL-1, MOLM-13 and MV4-11 but not SH-1. (TIF) [file pone.0240120.s002.tif]

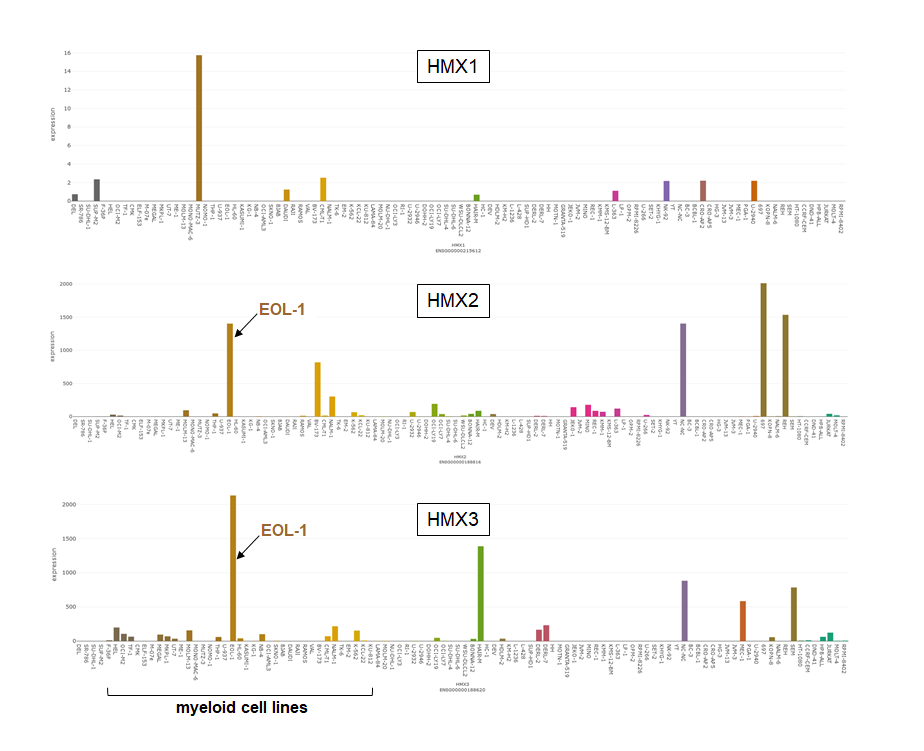

Supplement: S3 Fig — According to RNA-seq data from LL-100 and setting the cut-off at 500, these data show absent expression of HMX1 in all cell lines while HMX2 and HMX3 are active in selective cell lines from different origin. (TIF) [file pone.0240120.s003.tif]

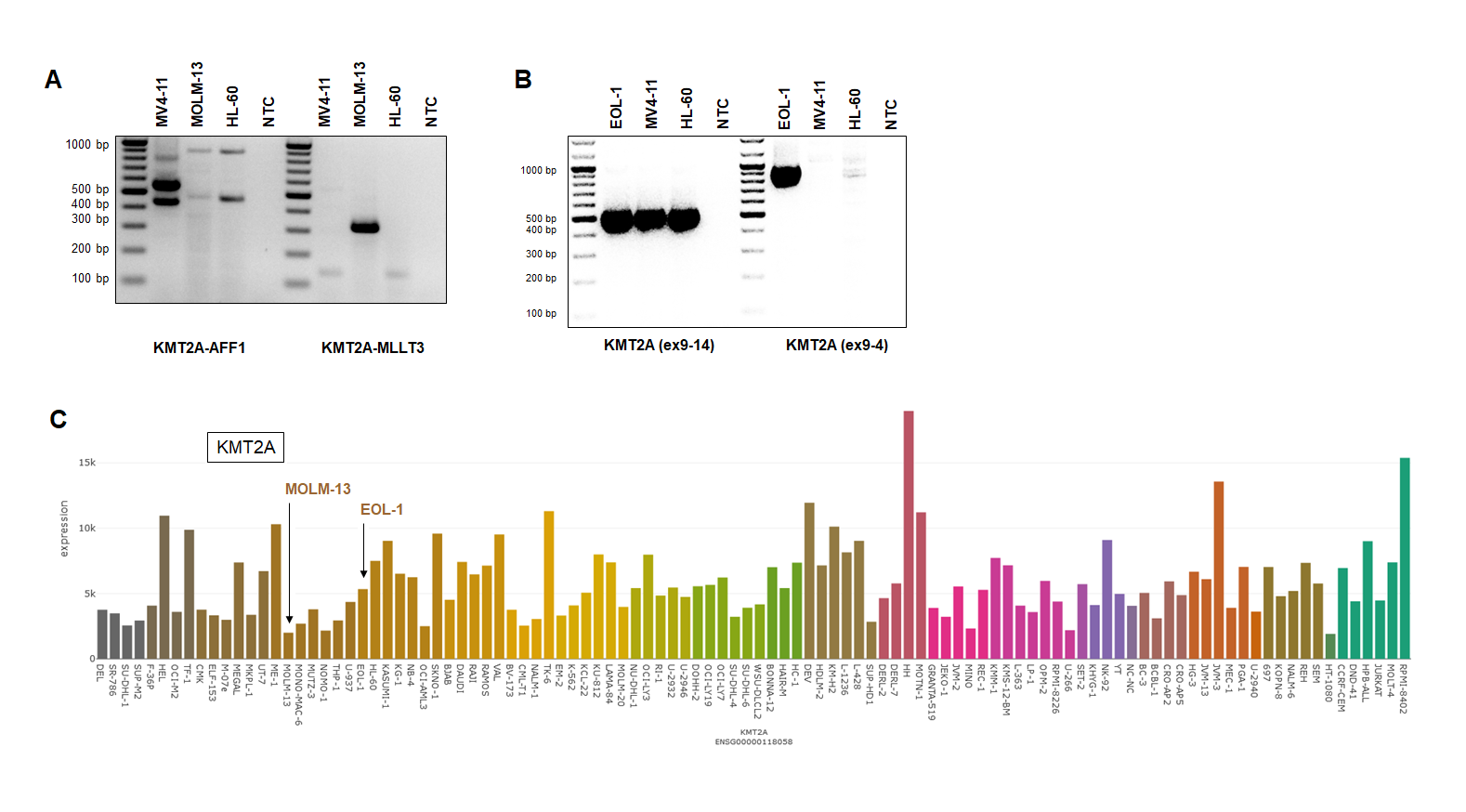

Supplement: S4 Fig — (A) RT-PCR analysis of KMT2A-fusions in selected AML cell lines demonstrates KMT2A-AFF1 in MV4-11 (left) and KMT2A-MLLT3 in MOLM-13 (right). NTC: no template control. (B) RT-PCR analysis in selected AML cell lines of KMT2A (left) and of KMT2A-PTD (right). (C) LL-100 data for KMT2A RNA expression. (TIF) [file pone.0240120.s004.tif]

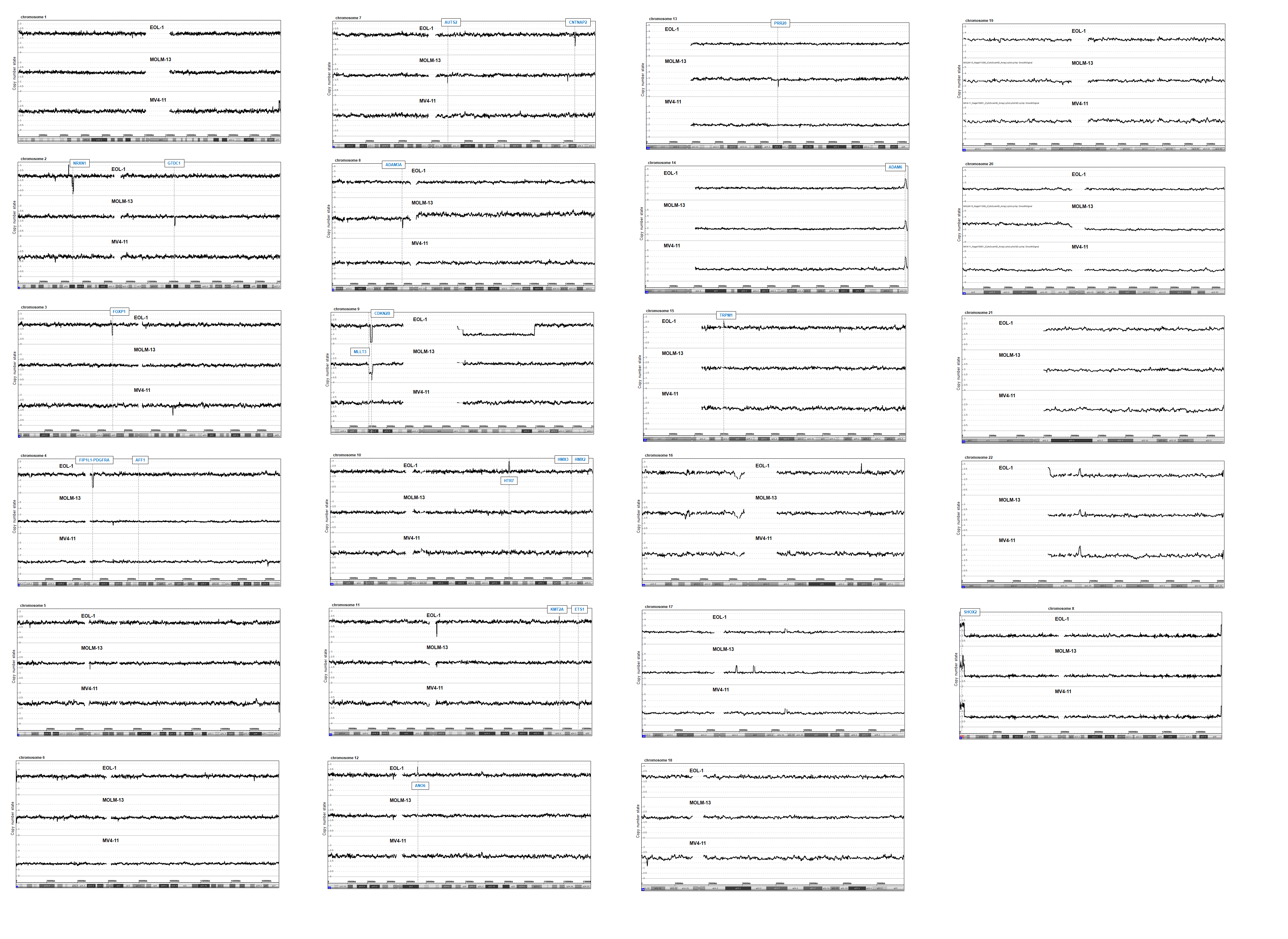

Supplement: S5 Fig — Genomic profiling shows copy number alterations at 4q12 (FIP1L1-PDGFRA), 10q23 (HTR7), and 11q23 (KMT2A) in EOL-1. EOL-1 and MOLM-13 share a deletion at 9p21 containing CDKN2B. In MOLM-13, this deletion is involved in ins(11;9)(q22;p23) generating fusion gene KMT2A-MLLT3. No aberrations were found at the HMX2/3 locus at 10q26. (TIF) [file pone.0240120.s005.tif]

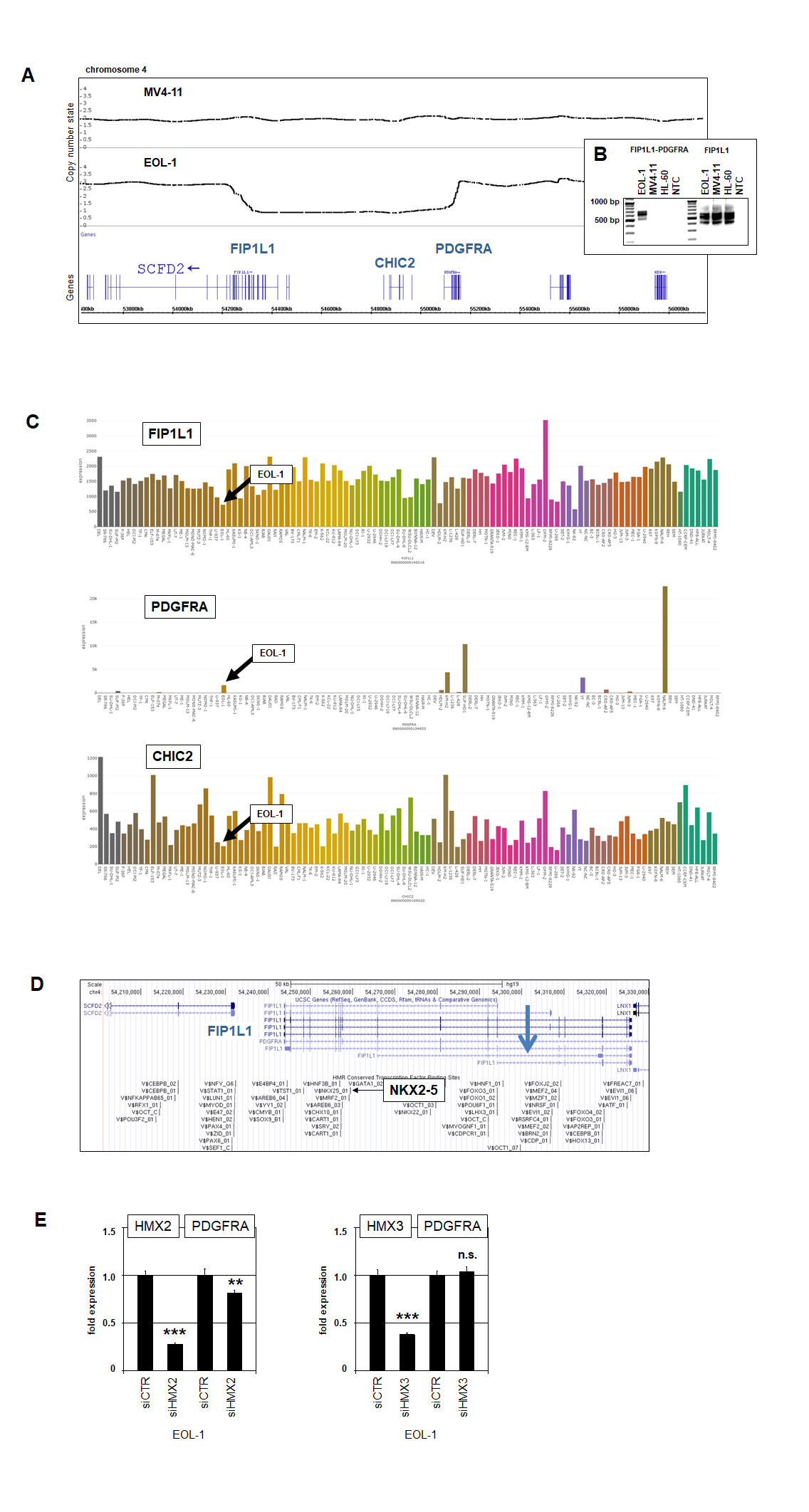

Supplement: S6 Fig — (A) Genomic profiling data show a deletion in EOL-1 at 4q12 which targets FIP1L1 and PDGFRA and removes CHIC2. (B) RT-PCR analysis of FIP1L1-PDGFRA (left) and of FIP1L1 (right) as control. (C) LL-100 data for FIP1L1, PDGFRA and CHIC2. (D) A genomic map of the locus for FIP1L1 was taken from the UCSC genome browser, showing potential transcription factor binding sites including a potential NKX2-5-site. (E) SiRNA-mediated knockdown of HMX2 (left) resulted in reduced expression levels of PDGFRA, indicating an activating impact while knockdown of HMX3 showed no alteration (right). (TIF) [file pone.0240120.s006.tif]

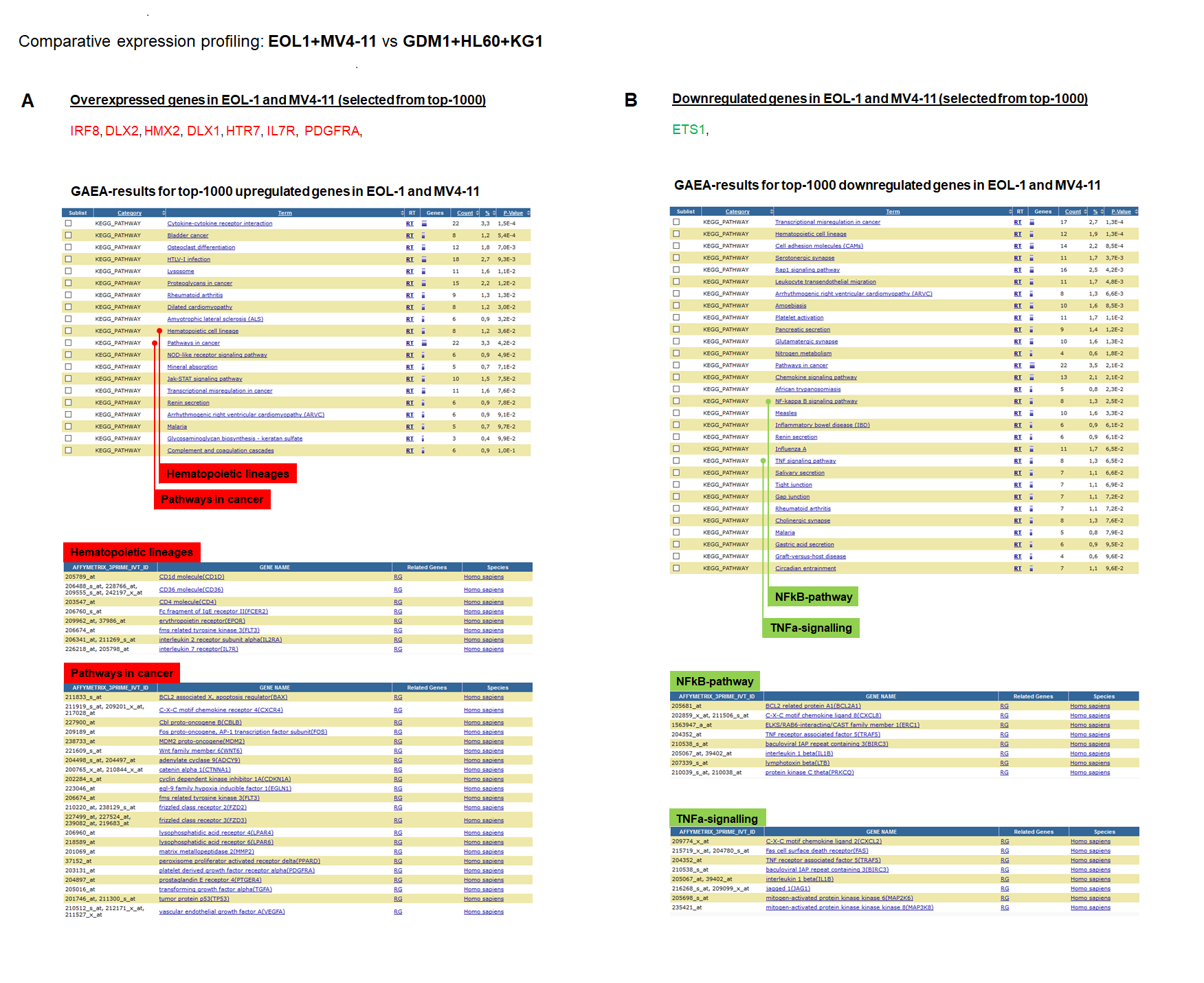

Supplement: S7 Fig — (A) Lists of differentially expressed genes in EOL-1 and MV4-11 as compared to the controls GDM-1, HL-60 and KG-1. Genes are arranged in the order of fold expression differences. (B) Gene-annotation enrichment analysis for AML cell lines EOL1 and MV4-11 using the top-1000 upregulated genes. Identified KEGG-pathways included JAK-STAT- and WNT-pathway. (C) Gene-annotation enrichment analysis for AML cell lines EOL1 and MV4-11 using the top-1000 downregulated genes. Identified KEGG-pathways included the NFkB-pathway. (TIF) [file pone.0240120.s007.tif]

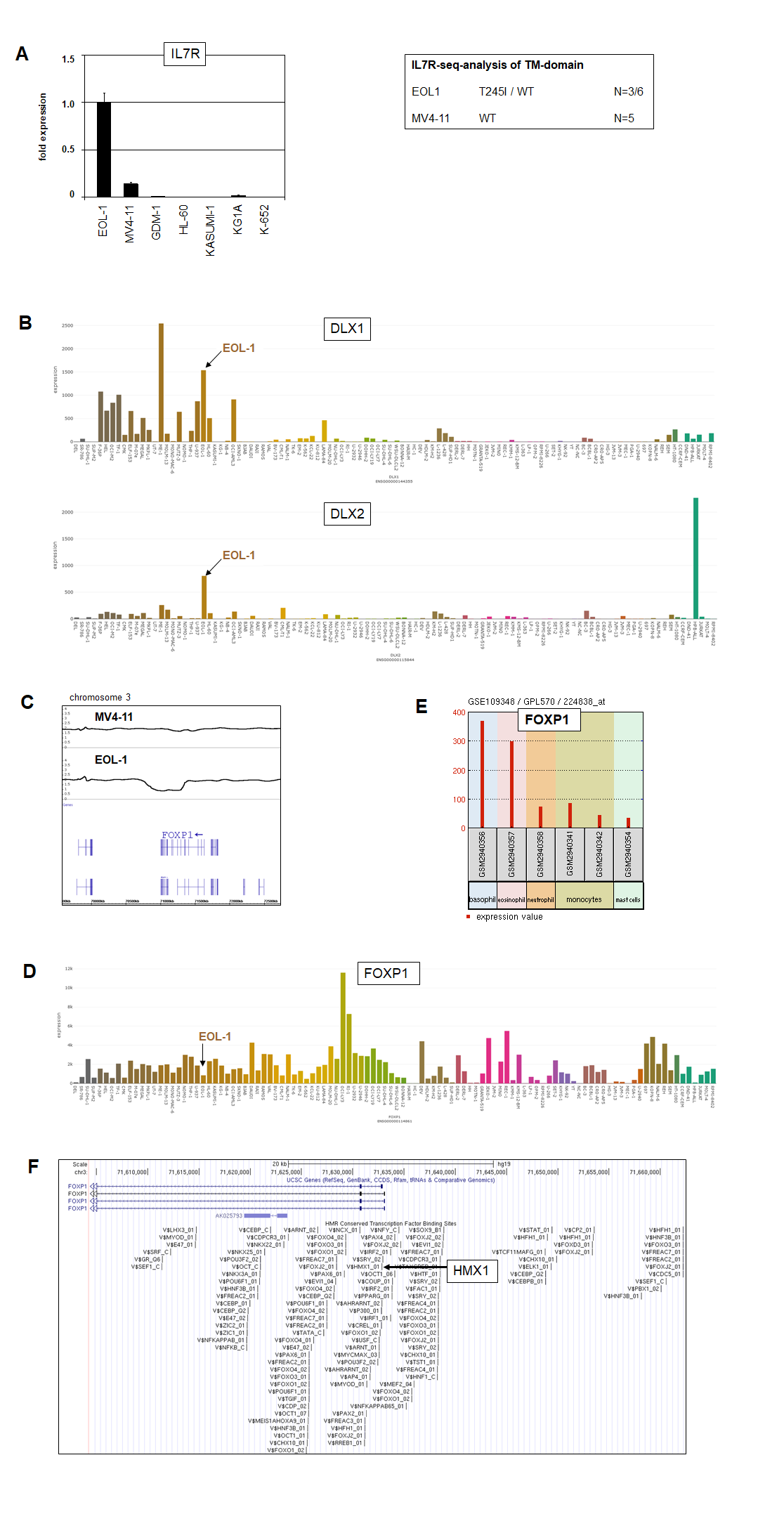

Supplement: S8 Fig — (A) RQ-PCR analysis of IL7R in selected AML cell lines (left). Sequencing results of cloned PCR products encompassing the TM-domain of IL7R (right). For MV4-11 we obtained five wildtype sequences, for EOL-1 we obtained three mutated and six wildtype sequences. (B) LL-100 data for DLX1 and DLX2 RNA expression. (C) Genomic profiling data show a deletion in EOL-1 at 3p13 which targets FOXP1. (D) LL-100 data for FOXP1 RNA expression. (E) FOXP1 expression data for primary cells obtained from dataset GSE109346. (F) A genomic map of the locus for FOXP1 was taken from the UCSC genome browser, showing potential transcription factor binding sites including a potential HMX1-site. (TIF) [file pone.0240120.s008.tif]

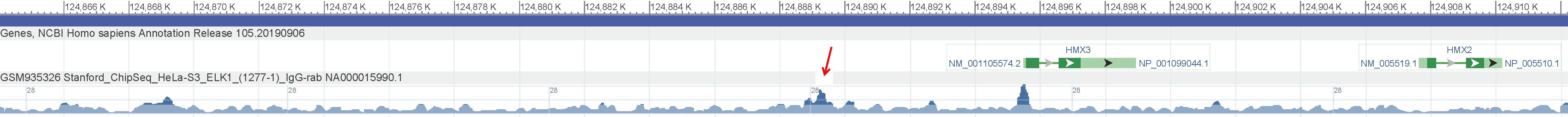

Supplement: S9 Fig — The peaks at the transcriptional start site and in the upstream region (red arrow, corresponding to the mutated site in EOL-1) indicate ELK1 interaction at the HMX-locus. (TIFF) [file pone.0240120.s009.tiff]

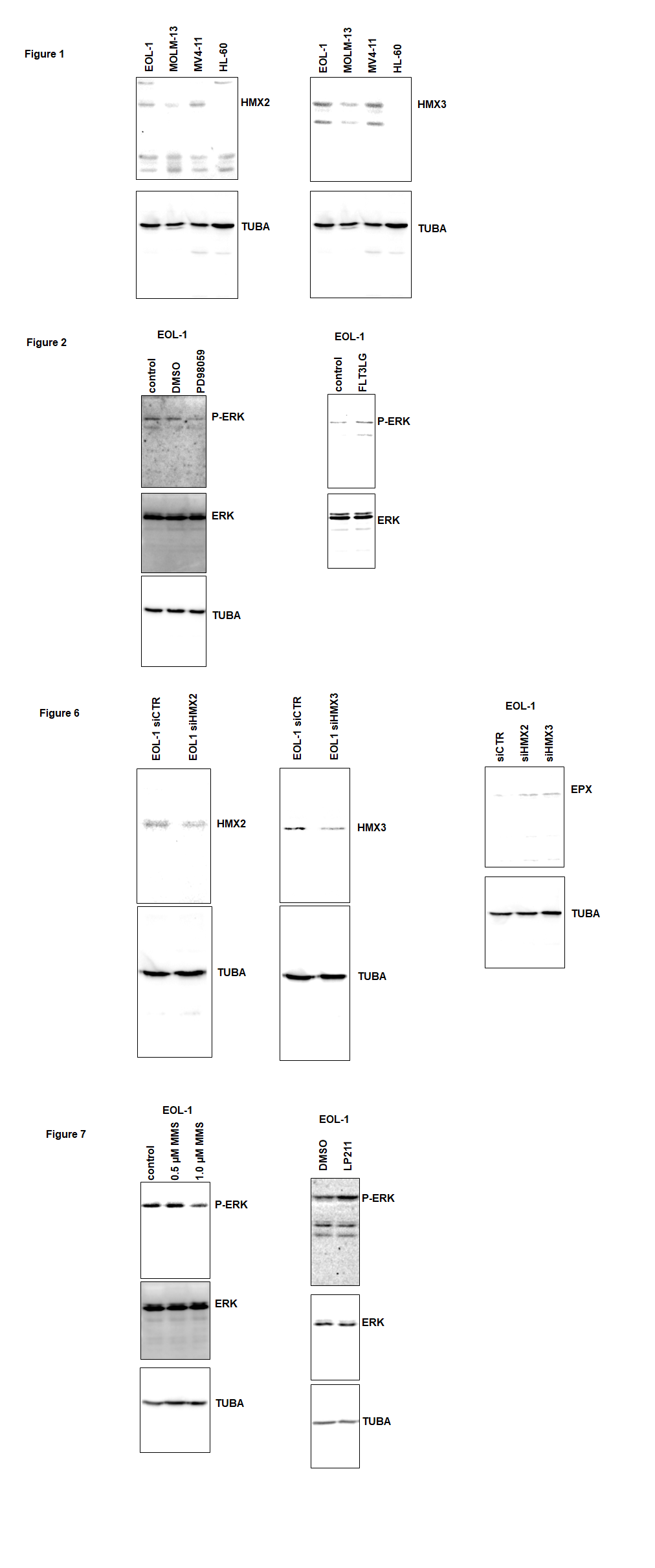

Supplement: S1 Raw images — (TIF) [file pone.0240120.s010.tif]
